# Evaluation of a Large-Scale School Wellness Intervention Through the Consolidated Framework for Implementation Research (CFIR): Implications for Dissemination and Sustainability

**DOI:** 10.3389/frhs.2022.881639

**Published:** 2022-04-28

**Authors:** Gabriella M. McLoughlin, Rachel Sweeney, Laura Liechty, Joey A. Lee, Richard R. Rosenkranz, Gregory J. Welk

**Affiliations:** ^1^College of Public Health, Temple University, Philadelphia, PA, United States; ^2^Implementation Science Center for Cancer Control and Prevention Research Center, Brown School, Washington University in St. Louis, St. Louis, MO, United States; ^3^4-H Extension and Outreach, Iowa State University, Ames, IA, United States; ^4^Department of Health Sciences, University of Colorado Colorado Springs, Colorado Springs, CO, United States; ^5^Department of Food, Nutrition, Dietetics and Health, Kansas State University, Manhattan, KS, United States; ^6^Department of Kinesiology, Iowa State University, Ames, IA, United States

**Keywords:** dissemination, implementation, sustainability, children, health promotion, obesity prevention, school wellness

## Abstract

**Background:**

Numerous studies have tested school-based interventions promoting healthy behaviors in youth, but few have integrated dissemination and implementation (D&I) frameworks. Using D&I frameworks can inform if and how an evidence-based intervention is implemented and maintained and provide strategies to address contextual barriers. Such application is necessary to understand how and why interventions are sustained over time. We evaluated a school wellness initiative called SWITCH® (School Wellness Integration Targeting Child Health) to (1) assess implementation outcomes of adoption, fidelity, and penetration, (2) discern implementation determinants through the Consolidated Framework for Implementation Research (CFIR), and (3) examine differences among inexperienced and experienced schools and influential factors to sustainment.

**Methods:**

A total of 52 schools from Iowa, United States enrolled in the 2019–2020 iteration of SWITCH (22 inexperienced; 30 experienced). The CFIR guided the adaptation of mixed methods data collection and analysis protocols for school settings. Specific attention was focused on (1) fidelity to core elements; (2) adoption of best practices; and (3) penetration of behavior change practices. Determinants were investigated through in-depth qualitative interviews and readiness surveys with implementation leaders. A systematic process was used to score CFIR domains (between −2 and +2) indicating positive or negative influence. Independent *t*-tests were conducted to capture differences between samples, followed by a cross-case analysis to compare determinants data. Inductive coding yielded themes related to sustainment of SWITCH beyond formal implementation support.

**Results:**

Experienced schools had higher scores on fidelity/compliance (*t* = −1.86 *p* = 0.07) and adoption (*t* = −2.03 *p* = 0.04). CFIR determinants of innovation source, culture, relative priority, and leadership engagement were positive implementation determinants, whereas tension for change and networks and communications were negative determinants. Distinguishing factors between experienced and inexperienced schools were Readiness for Implementation and Self-efficacy (experienced significantly higher; *p* < 0.05). Strategies to enhance sustainability were increasing student awareness/advocacy, keeping it simple, and integrating into school culture.

**Conclusions:**

Findings provide specific insights related to SWITCH implementation and sustainability but more generalized insights about the type of support needed to help schools implement and sustain school wellness programming. Tailoring implementation support to both inexperienced and experienced settings will ultimately enhance dissemination and sustainability of evidence-based interventions.

## Introduction

School-based health promotion interventions have been shown to have a positive impact on promoting student physical activity and nutrition behaviors (1–5); however, systematic application of dissemination and implementation science (D&I) frameworks are needed to advance the gap between research and practice (6, 7). Furthermore, despite the promise of comprehensive programs, limited research exists to illustrate steps to sustain programs over time (8, 9). Particular emphasis is needed to evaluate strategies aimed at building capacity in school systems since programming is a shared responsibility. Without guidance on how to sustain interventions, school leaders are likely to abandon programming over time, leading to diminished impacts on children's health and well-being.

The present paper reports on the capacity-building process employed in a school wellness initiative called SWITCH® (School Wellness Integration Targeting Child Health). The initiative was built on the foundation of an evidence-based obesity prevention program called Switch that worked through schools to help students “*switch what they do, view, and chew*” (10–12). Through a United States Department of Agriculture (USDA) grant, emphasis shifted to building capacity in schools to independently coordinate and sustain school wellness programming based on Switch. Formalized D&I strategies were critical in facilitating the transition from an evidence-based program (i.e., Switch) to an evidence-based process (i.e., SWITCH) for sustaining health promotion in schools. Schools self-enroll in a cyclical training (Fall) and implementation (Spring) process which prepares them to develop a comprehensive approach to student health promotion (physical activity, screen time, nutrition behaviors). The process is aimed at helping schools to meet mandates such as the USDA final rule, which tasks schools with developing and evaluating school wellness programs and policies (13, 14).

Foundational research by our team documented the feasibility of training school leaders (15), the acceptability of educational modules for classroom, physical education, and lunchroom settings (16–18) and the validity of school readiness and wellness environment assessment tools (19, 20). Subsequent studies evaluated alternative implementation strategies (21), the levels of engagement by 4-H leaders (county-level Extension officers who facilitate local-level implementation) assisting in programming (22) and the factors that influenced implementation and scale-up (13). This most recent evaluation focused on capacity-building and highlighted changes in organizational readiness, reflecting prior literature and warranting its inclusion in subsequent evaluation (23–26). Guided by D&I principles, SWITCH programming has transitioned to be fully managed and coordinated by leaders within the state 4-H network who lead local-level programs and initiatives (https://www.iowaswitch.org/). The established infrastructure provides an ideal model to understand the factors influencing implementation and sustainability of school wellness programming.

The Consolidated Framework for Implementation Research (CFIR) (27, 28), referred to as a *determinants framework* in the D&I literature, offers specific advantages for a more comprehensive analyses of factors influencing implementation of SWITCH. Specifically, CFIR comprises 39 constructs housed within six key domains: Intervention Characteristics (factors within the intervention itself such as cost and complexity); Outer Setting (factors external to the implementation setting such as policy); Inner Setting (factors within organization such as networks, culture); Readiness for Implementation (organizational and individual capacity for implementation); Characteristics of Individuals (implementation leaders' confidence and motivations to implement); and Implementation Process (practices that facilitate implementation such as planning and executing). Such framework has been used predominantly in healthcare settings to investigate determinants of implementation (28–31), with growing application to school and community settings (32, 33). The CFIR website (www.CFIRguide.org) provides comprehensive resources for researchers conducting qualitative and mixed methods evaluation to ground their analysis through systematic coding of interview/qualitative data to facilitate interpretation (31). The CFIR constructs guided several recent mixed method studies on the 2018–2019 iteration of SWITCH (13); however, it was not possible to fully integrate the interview and implementation outcome data and this hindered our ability to understand determinants that linked to specific implementation outcomes.

The present study on the 2019–2020 iteration of SWITCH employs an integrated mixed methods analysis, based on CFIR coding methods (23), to better understand the factors that influence implementation and sustainability of school wellness programming. The CFIR methodology has documented utility for clinical research (23, 28, 31), but this is one of the first systematic applications of CFIR mixed methods analysis methods for evaluating programming in community / school settings. The study builds directly on our past work (13) by seeking to understand the factors that explain variability in implementation effectiveness between experienced and inexperienced schools. Readiness for implementation has been identified as a barrier to *sustaining* evidence-based interventions in schools (9); but few studies have directly examined the relationships between implementation determinants (such as readiness) and outcomes in school-based health promotion research (34, 35). Addressing this gap was the main goal of the 2019–2020 iteration of SWITCH. Accordingly, this study had three primary aims:

1) To assess implementation outcomes of adoption, fidelity, and penetration of SWITCH.
2) To discern implementation determinants grounded in the CFIR through a deductive approach.
3) To examine the differences in outcomes and determinants among new and experienced schools, and influential factors to sustainment of SWITCH.

Results of this study will provide critical information which may help inform implementation strategies for scale-up and sustainability in school-based interventions.

## Materials and Methods

A mixed methods implementation study grounded in the CFIR was conducted to evaluate key outcomes, determinants, and nuanced relationships between these factors among new and experienced schools in the 2019–2020 cycle of SWITCH. Evaluation approaches followed recommended data collection and analytic methodologies of CFIR, developed by Damschroder and colleagues (27, 31). To our knowledge, this is one of the first documented adaptations of the CFIR mixed methods protocols with the goal of understanding relationships between implementation determinants and outcomes within a school health promotion context.

### Participants and Procedures

A total of 52 schools enrolled in the 2019–2020 iteration of SWITCH (30 had prior experience and 22 had no previous exposure). Demographic information for these schools is shown in Table 1. The cyclical training (fall) and implementation (spring) process of SWITCH across the academic year facilitates a continuous quality improvement process (36), whereby feedback from schools and implementation outcome data drive modifications to the program each year. More information about the training process can be found in Additional File 1, our previously published article (13), and the program website (https://www.iowaswitch.org/). Briefly, schools were asked to form a wellness team which comprised three members of staff across different school settings (e.g., classroom teachers, physical education, food service, other teachers, administration, counselors, nurses, etc.) and to register prior to the beginning of the academic year. Following registration, schools were asked to attend a total of four webinars and an in-person conference during the fall semester, as well as complete several pre-program audit tools. The implementation phase spanned a 12-week period from January–April of 2020, but due to the coronavirus (COVID-19) outbreak, schools were forced to close in Iowa on March 13th thus forcing a transition to virtual communications/implementation after week 8 of the program. It was not possible to capture final outcome data, but schools completed the midpoint evaluation of school implementation. Below we outline data sources for implementation outcomes and determinants, and the steps taken to rigorously analyze these data.

**Table 1:** School demographic information for the 2019–2020 Cohort.

	**Free/reduced meals (%)**		**Racial/ethnic minority (%)**		**Enrollment**		**Experience (years)**	
	**Mean**	***SD***	**Mean**	***SD***	**Mean**	***SD***	**Mean**	***SD***
Total (*n* = 52)	49.1	19.0	15.4	18.6	226.3	180.1	1.8	0.8
Inexperienced (*n* = 22)	50.7	21.9	20.0	21.5	224.6	200.5	NA	NA
Experienced (*n* = 30)	48.0	17.2	12.1	15.9	227.5	168.0	2.47	0.57

*Free/reduced meals, percentage of students eligible for free/reduced meals; experience, years of experience in the program including present year (range 1–4). NA, not applicable*.

### Measurement of Implementation Outcomes: Adoption, Fidelity, and Penetration

The field of D&I offers many frameworks and theories to help researchers and practitioners discern why evidence-based practices are or are not implemented in routine care. Regarding *implementation outcomes* frameworks, the framework by Proctor and colleagues (37) conceptualized several distinctive outcomes that are important to include within implementation evaluations: (1) acceptability (the degree to which an innovation is a perceived good fit); (2) adoption (intent to implement); (3) appropriateness (degree of compatibility within setting); (4) cost (to implement, value for money); (5) feasibility (possibility of successful implementation); (6) fidelity/compliance (executed as intended); (7) penetration (reach within setting); and 8) sustainability (long-term impact). For the purpose of this study, we chose to examine the determinants of adoption, fidelity, and penetration among schools enrolled in SWITCH due to the heavily integrated implementation practices needed to create systems change in the school setting.

Adoption is operationalized by Proctor and colleagues (37) as “intention, initial decision, or action to try or employ an innovation or evidence-based practice” (p. 69). Thus, we measured adoption through implementation surveys at the 6-week mark, examining uptake of best practices in various settings (use of curricular modules, posters, reinforced themes through discussion and tracking). Each best practice was scored as 0 (not at all implemented), 2 (somewhat implemented), and 3 (fully implemented) and a summed score was generated based on the average of each component, to give possible range of 0–9.

Fidelity relates to “the degree to which an intervention was implemented as it was prescribed in the original protocol or as it was intended by the program developers” (p. 69) (37). The quality elements of SWITCH comprise; wellness team meeting (ideally at least once per week); using SWITCH website to promote student behavior tracking; engaging parents and other stakeholders; and integration of SWITCH modules/posters across the school setting. Fidelity therefore was calculated by using a summed score of quality elements which were scored the same way as best practices, giving a possible range of 0–12.

Finally, penetration is defined as the “integration of a practice within a service setting and its subsystems” (p.70) (37). This was calculated by determining the number of participants who used or interacted with an evidence-based practice, divided by the total number of participants eligible or within the sample. Since the behavioral tracking and goal setting interface is an integral component for students (38), it provides a good indicator of how many students are actively engaged in SWITCH within each school, thus providing data on penetration. We used data from SWITCH behavior tracking across weeks 1–8 (to account for COVID-19-related school closures). These data are presented as a decimal score (range 0–1.0, translated to 0–100%).

## Implementation Determinants

### Organizational Readiness

The School Wellness Readiness Assessment (SWRA) tool (20) was used to assess baseline readiness for implementation. Developed in line with the theory of organizational readiness for change (26, 39) and community capacity-building frameworks (40), the SWRA captures the unique, complex structure and specific settings within schools that impact student health, including classrooms, physical education, and lunchroom settings, and the broader school leadership and cultural context.

The SWRA includes questions across four subscales designed to assess setting-specific and school-wide wellness readiness: classroom readiness, physical education (PE) readiness, food services readiness, and school readiness. The SWRA items were assessed using a 5-point scale (strongly disagree, disagree, neither agree nor disagree, agree, and strongly agree scale, coded as 0, 1, 2, 3, and 4, respectively). A copy of the SWRA is provided in Additional File 2. Wellness teams completed the 40-item SWRA through the program website. Scores for each of the subscales were calculated by averaging together the item responses in each section with higher scores representing higher states of readiness in specific settings and schools.

### Qualitative Interviews Grounded in CFIR

Following procedures developed by Damschroder and colleagues (28, 31, 41), an interview guide was developed which aimed to understand the influence of each CFIR domain on implementation of SWITCH (see Additional File 3). Each school's wellness team was invited to participate and we asked as many people as possible to attend the interviews (usually 3 per team). Questions were open-ended; examples included, “What is your perception of the quality of the modules, posters, and other SWITCH materials that were provided?” (Innovation Characteristics – Design Quality and Packaging) and “How do you think your school culture affected the implementation of SWITCH programming?” (Inner Setting – Implementation Climate). Interviews were conducted by a qualitative and survey methodologist to ensure impartiality in responses from school wellness teams. To address issues of sustainability, interviewers asked “Think of the changes you have made in your school setting. To what degree do you think these changes are sustainable?” and then prompted participants to expand on their responses with examples. The goal was to encourage candid responses so time limits were not imposed on these conversations. This ensured in-depth understanding of each context and implementation climate.

Of the 52 schools enrolled in SWITCH, 45 (87% of sample) completed interviews. Of these 45, 17 were new and 28 were experienced. Each school that participated had between 1 and 3 members of their school wellness team present. Table 2 shows representation of the various school staff positions within school wellness teams and those who were present in interviews; classroom and physical education teachers were included in most wellness teams and were most present on interviews, followed by food service and principals. Interviews lasted between 31 and 63 min, were conducted through video conferencing software (i.e., Zoom), and transcribed verbatim.

**Table 2:** Representation of various staff members in the total sample, then split by experience level.

**School staff role**	**# Represented in total sample** ***N*** **=** **52 schools**		**# Represented in inexperienced schools** ***n*** **=** **22**		**# Represented in experienced schools** ***n*** **=** **30**	
	**Wellness teams**	**Interview *n* = 45**	**Wellness teams**	**Interview *n* = 17**	**Wellness teams**	**Interview *n* = 28**
Classroom teacher	59	21	22	7	37	14
Counselor	5	2	5	2	0	0
Food service/nutrition	17	3	7	2	10	1
Instructional coach	5	3	2	1	3	2
Nurse	15	4	3	0	12	4
Paraprofessional	1	0	0	0	1	0
Physical education	32	13	13	3	19	10
Principal	19	7	10	4	9	3
Superintendent	1	0	0	0	1	0
Other	9	2	4	1	5	1

*Sample size in top row refers to total number of schools in each group; numbers in cells represent the total representation of specific roles on lead wellness teams and present in interviews. Total numbers can exceed the number of schools due to size of wellness teams*.

### Qualitative Data Coding and Case Memos

The structure of the interview guide facilitated a predominantly deductive data analysis approach, in that each of the questions corresponded to a construct within each of the framework domains (31). However, we remained open, such that any themes that emerged through inductive approaches were included in our analyses; such combination of deductive and inductive coding integrates data-driven codes with theory-driven ones (42). For example, for the interview questions that addresses sustainability (additional files), we coded data from these responses deductively where they aligned with relevant constructs of CFIR but also inductively to provide critical information to the research team on what factors influence sustainment.

First, the lead and second author met to develop a coding consensus document (Additional File 4), which described each CFIR construct and anticipated potential responses and themes that would emerge through the data. Applying the CFIR systematic coding approach facilitated the assignment of numerical scoring to the qualitative data, such that if a particular construct was deemed to have a positive influence on implementation based on interview responses, a score of +1 or +2 was assigned for that construct. Conversely, if a construct was deemed to be a negative influence, a score of −1 or −2 was given. If it was not clear whether a positive/negative influence manifested, a score of 0 was given; a score of “X” was used for mixed results (see Additional File 5 for details on CFIR rating rules) (31).

Second, to establish inter-rater reliability, the two coders selected five transcripts and created independent case memos using the CFIR memo templates (41). Scores were compared and a percent agreement score was calculated; if the overall agreement score was <80%, the coders met to ensure consensus before coding another set of five transcripts. Once ≥80% agreement was met, the second author coded the remaining transcripts, before a randomly selected set of five transcripts was reviewed by the lead author. All coding was completed in memo documents (see Additional File 6). Finally, to facilitate content analysis and interpretation of trends in interview data, all memos were entered in to NVivo qualitative analysis software and coded into respective nodes, following the CFIR codebook template (41). To prepare the quantified CFIR data for merging into the larger dataset, each school ID was aligned with the scores for each construct and domain of the model. Any X scores (implying a mixed/uncertain rating) were converted to 0 for the purpose of analysis. Any scores without a score remained blank so as not to misguide subsequent analyses.

## Data Analysis

### Aims 1 and 2: Evaluate Outcomes and Determinants

All school demographic, implementation outcome, and quantified implementation determinant data from the coded CFIR interviews were merged using SAS software (Version 9.4, Cary NC) to facilitate descriptive and inferential analyses. First, descriptive tests were conducted to obtain means (and SD) for all implementation outcome and determinant data, then split by experience level (0 = inexperienced; 1 = experienced). Following recommendations from Damschroder et al. (23) Pearson bivariate correlations were run to establish correlations between implementation outcomes and determinants to examine associations and to understand potential influences of implementation for schools that experienced greater success. All tests were run in SAS software (Cary, NC), and α significance was assumed as *p* < 0.05; correlations with *p* < 0.10 were also highlighted due to the novel nature of this work. Based on such associations, salient quotes from interview transcripts were extracted to provide rich contextual details on determinants.

### Aim 3: Investigate Nuanced Determinants for New and Experience Schools

To investigate distinguishing factors among inexperienced and experienced schools, an in-depth cross-case analysis was conducted based on prior evaluations through the CFIR in other settings (30, 31, 43, 44). Cross-case analysis provides a broad scope for researchers to systematically compare multiple “cases” (i.e., schools) and is a derivative of Qualitative Comparative Analysis (QCA) (45, 46). We pursued a combination of exploratory analysis and cross-case analysis to investigate the distinguishing factors between experienced and inexperienced schools. Given that our sample size afforded exploratory inferential testing, we first conducted independent *t*-tests to examine differences in mean scores for each CFIR construct between the two samples (α significance was assumed as *p* < 0.05). In addition, we sought constructs which had >0.5 difference in mean score between the two samples, to highlight other distinguishing factors which may influence implementation (31). Subsequently, the research team explored qualitative extracts using NVivo as a means to contextualize findings from correlation analyses. Such an approach allowed for deeper contextual understanding of implementation practices which triangulate implementation determinants and outcomes (13).

To establish credibility, dependability, and trustworthiness, three key steps were taken in the analyses (47, 48). First, although the coding methods applied a deductive process, the lead researcher regularly conducted peer debriefing with other members of the research team to minimize potential bias and assumptive coding. Second, the mixed methods design facilitated methods triangulation throughout analysis procedures which ensured that distinguishing factors gleaned through cross-case analysis were properly contextualized and refuted if not enough substantive evidence existed (49). Finally, the use of coding memos provided the researchers with a method of maintaining an audit trail while coding, in which they took rigorous notes. This was exceptionally useful when establishing inter-rater reliability.

## Results

### Aim 1: Implementation Outcomes

**Figure 1:**
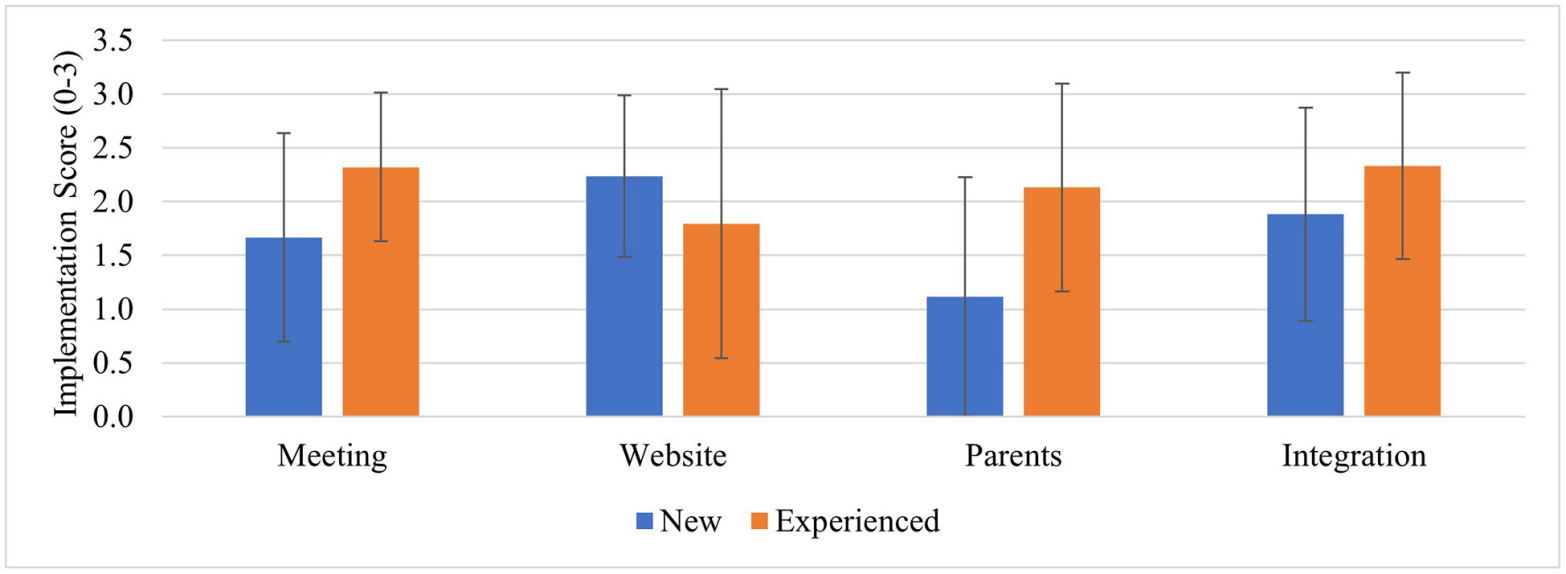
Fidelity to SWITCH quality elements (Mean, SD) by experience level. Checkpoint surveys conducted at week 6; Implementation fidelity scores 0, not implemented at all, 2, somewhat implemented, 3, implemented fully; meeting, school wellness team meeting; website, setting up classrooms and student tracking in the website; parents, parent outreach activity; integration, implementing educational modules/resources across each of the SWITCH settings.

Schools reported strong fidelity (mean score 7.6 ± 2.91); however, this varied by schools and by item. Experienced schools reported better fidelity overall except for using the SWITCH website (see Figure 1). Parent outreach was the lowest implemented practice; outreach activities mostly entailed sending newsletters that were provided by the SWITCH team (70%); experienced schools reported more parent outreach practices than inexperienced schools. The most common method of school-wide integration was sending emails to the staff to inform them of the program and activities (88%) followed by using posters to promote SWITCH themes in different settings (73%). For adoption (5.53 ± 2.17), experienced schools reported significantly higher rates according to independent samples *t*-tests (*t* = −2.03, *p* = 0.04). This difference was consistent across use of modules, posters, and tracking/reinforcing themes. The highest implemented practice was classroom tracking followed by tracking in physical education setting (see Additional File 7).

Regarding penetration, behavioral tracking data demonstrate that inexperienced and experienced schools were approximately equal in terms of tracking rates between week 1 and week 7; 43 ± 29% of students in inexperienced schools and 46 ± 32% of experienced schools tracked each week (mean score 0.448, or 45%). Tracking naturally dropped due to COVID-19-related school closures but it is noteworthy that rates were essentially 0% for inexperienced schools but 25% for experienced schools. This indicates that the experienced schools were more likely to retain tracking rates to a greater extent than inexperienced schools. Only data from the first 8 weeks are used for the related correlation analyses.

### Aim 2: Implementation Determinants

**Figure 2:**
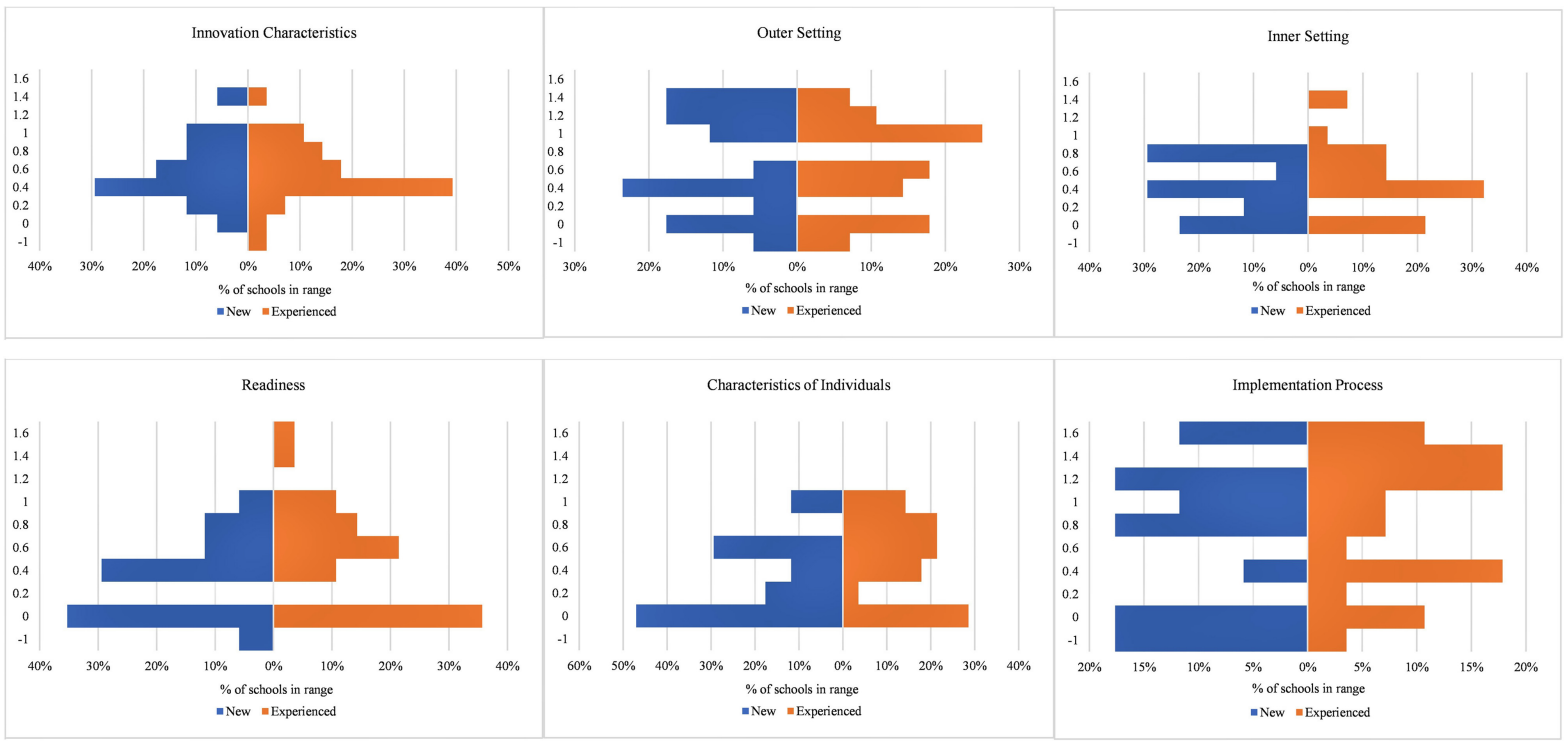
Dual-sided histogram of CFIR domain scores, by experience level. Graph shows percentage of schools falling in specific ranges for global domain score (Y axis): −1 to −0.5; −0.5 to 0; 0 to 0.2; 0.21 to 0.4; 0.41 to 0.6; 0.61 to 0.8; 0.81 to 1.0; 1.01 to 1.2; 1.21 to 1.4; 1.41 to 1.

The process of converting qualitative interview data to numerical scores through CFIR protocols facilitated our ability to detect factors that were influential to SWITCH implementation outcomes. However, analysis of Cronbach's alpha revealed that none of the CFIR domains had acceptable internal consistency (all < 0.40). We therefore felt it important to show variability in the data as opposed to means and SD of global domains. Figure 2 displays scores from each domain as dual-sided histograms to facilitate examination of variability, separated by experience level (discussed below). From examination it appears that for all schools, factors within the Outer Setting and Implementation Process domains were most positively ranked, but high variability must be noted.

Table 3 displays all means ± SD for CFIR construct data. In terms of positive influential factors, data reveal that the most positive scores from coding of interview data were Readiness for Implementation – Leadership Engagement (i.e., building administration involvement/support; mean = 1.22 ± 1.02), Individual Characteristics – Knowledge and Beliefs about the Intervention (i.e., school wellness teams' perceptions of SWITCH; 1.51 ± 0.51), and Implementation Process – External Stakeholders (i.e., county 4-H Extension officer support; 1.42 ± 1.12). Regarding negative influences, lowest scores were assigned to Inner Setting – Relative Priority (i.e., priority given to SWITCH over other programs; −0.31 ± 1.26), Readiness for Implementation – Available Resources (i.e., time, personnel, equipment; −0.96 ± 0.88), and challenges in Implementation Process - Key Stakeholders (i.e., engaging parents; −0.22 ± 1.31).

**Table 3:** CFIR coding results by school experience and distinguishing factors.

**CFIR Domain**	**Construct**	**Inexperienced**		**Experienced**		**Total**			***t***	**Cohen's *d***	***p***
		**Mean**	***SD***	**Mean**	***SD***	**Mean**	***SD***	***>0.5 difference***			
Intervention characteristics	Innovation source	0.41	0.71	0.21	0.57	0.29	0.63				
	Evidence strength and quality	1.06	0.90	0.79	0.88	0.89	0.88				
	Relative advantage	0.12	0.49	0.11	0.42	0.11	0.44				
	Adaptability	0.71	0.69	0.96	0.88	0.87	0.81				
	Trialability	0.00	0.00	0.07	0.66	0.04	0.52				
	Complexity (reverse)	0.06	1.03	0.43	0.92	0.29	0.97				
	Design quality	0.71	0.85	0.25	1.27	0.42	1.14				
	Cost (reverse)	0.41	0.51	0.64	0.87	0.56	0.76				
Outer setting	Student needs and resources	0.41	0.71	0.75	0.84	0.62	0.81				
	Cosmopolitanism	0.53	1.23	0.93	1.18	0.78	1.20	*			
	Peer pressure	0.71	1.05	0.25	0.70	0.42	0.87	*			
	External policy and incentives	1.06	1.14	0.68	0.94	0.82	1.03				
Inner setting	Structural characteristics	0.00	1.00	0.39	1.23	0.24	1.15				
	Networks communications	0.53	1.37	0.43	1.26	0.47	1.29				
	Tension for change	0.29	0.59	0.32	0.61	0.31	0.60				
	Relative priority	−0.41	0.94	−0.25	1.43	−0.31	1.26				
	Culture	0.88	0.86	0.82	0.94	0.84	0.90				
	Compatibility	0.76	1.15	1.32	0.67	1.11	0.91	*			
	Organizational incentives and rewards	0.00	0.00	0.07	0.26	0.04	0.21				
	Goals and feedback	0.53	0.80	0.43	0.57	0.47	0.66				
Readiness for implementation	Readiness for implementation	−0.12	1.22	0.89	0.74	0.51	1.06	**	−3.09	1.003	.005
	Learning climate	−0.12	0.33	0.14	0.52	0.04	0.47				
	Leadership engagement	1.35	1.06	1.14	1.01	1.22	1.02				
	Available resources	−1.24	0.83	−0.79	0.88	−0.96	0.88				
	Access to Knowledge and Information	0.65	1.46	0.36	1.16	0.47	1.27				
Individual characteristics	Knowledge and beliefs about intervention	1.35	0.49	1.61	0.50	1.51	0.51				
	Self-efficacy	−0.24	1.35	0.61	0.92	0.29	1.16	**	−2.50	0.731	0.01
	Individual stage of change	−0.06	0.24	−0.07	0.54	−0.07	0.45				
	Individual identification with organization	0.00	0.00	−0.07	0.66	−0.04	0.52				
	Other personal attributes	0.41	1.12	0.25	1.08	0.31	1.08				
Implementation process	Planning	0.29	1.05	0.21	1.42	0.24	1.28				
	Implementation leaders	0.88	1.11	0.79	0.74	0.82	0.89				
	Engaging	0.47	1.50	1.00	1.15	0.80	1.31	*			
	Opinion leader	0.24	1.39	0.54	1.07	0.42	1.20				
	Champions	0.94	0.90	1.07	1.02	1.02	0.97				
	External change agents	1.24	1.15	1.54	1.10	1.42	1.12				
	Key stakeholders	−0.47	1.18	−0.07	1.39	−0.22	1.31				
	Innovation participants	1.24	1.09	1.75	0.44	1.56	0.78	*			
	Executing	0.59	1.12	0.86	1.01	0.76	1.05				
	Reflecting and evaluating	0.12	0.33	0.25	0.65	0.20	0.55				

CFIR, Consolidated Framework for Implementation Research; reverse, items reverse coded; all items scored on a range from −2 to +2; * Distinctively different (>0.5 score difference); ** Statistically significantly different; for all definitions, please see coding consensus document- additional files.

*Cohen's d calculation: https://www.socscistatistics.com/effectsize/default3.aspx*.

**Figure 3:**
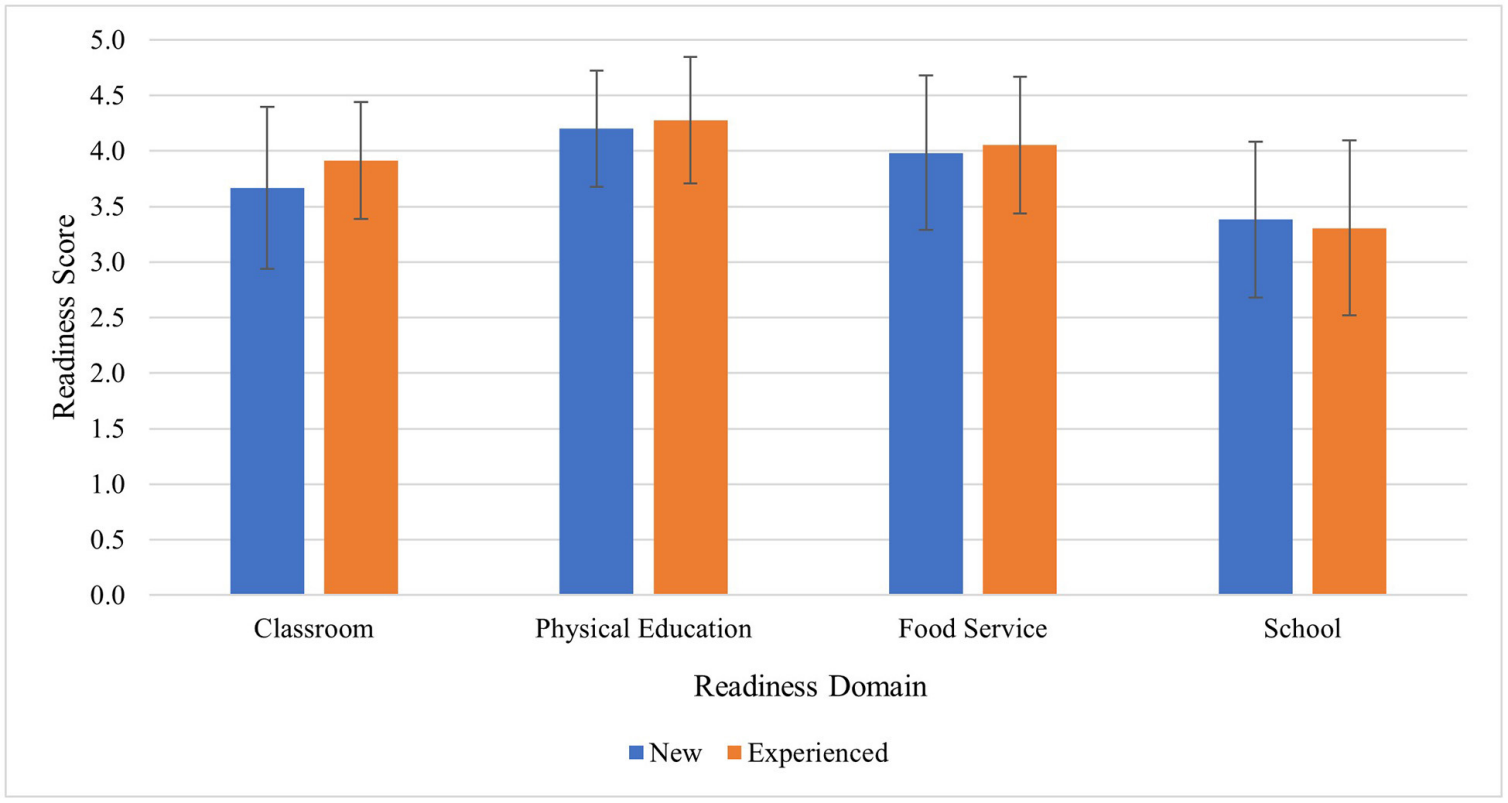
Baseline readiness scores (mean, SD) from the SWRA tool. Raw scores ranged from 1–5 (higher score, stronger readiness/organizational capacity), then separated into new and experienced schools.

Table 4 illustrates the results from exploratory Pearson bivariate correlation analyses for the whole sample. Almost all associations were positive, except Inner Setting-Networks and Communications (*r* = −0.28; *p* = 0.07) and Tension for Change (*r* = 0.27; *p* = 0.09), both negatively correlated with Adoption. Tension for Change was also negatively associated with Penetration (*r* = −0.33; *p* = 0.02). Salient interview extracts which relate to implementation outcomes are available in Additional File 8 and provide context for the whole sample. Figure 3 displays findings from the SWRA tool to assess baseline readiness/capacity. For the overall sample, a significant correlation was found between classroom readiness and adoption (*r* = *0*.366, *p* = 0.02). This illustrates that schools that reported greater classroom capacity were also using modules, tracking, and using posters more often than schools with lower classroom capacity. For the inexperienced schools, overall school capacity was positively correlated to adoption, indicating that organization-level readiness was associated with use of best practices across the school (*r* = *0*.513, *p* = 0.04). The lack of relationship between other capacity indicators and implementation outcomes is potentially due to the lack of variability in the capacity means. School and Class capacity had the largest range in scores (2 or 2.25 to 5) compared to PE and lunch capacity (between 3 and 5).

**Table 4:** Correlation analyses among determinants and outcomes.

**CFIR Domain**	**Construct**	**All schools (*****n*** **=** **47)**		
		**Penetration**	**Fidelity**	**Adoption**
Intervention characteristics	Innovation source	0.05	0.43**	−0.110
	Evidence strength and quality	−0.03	0.13	0.09
	Relative advantage	0.02	−0.04	−0.04
	Adaptability	0.15	0.03	−0.05
	Trialability	0.05	0.26	−0.11
	Complexity (reverse)	0.07	0.32**	0.08
	Design Quality	0	0.02	0.09
	Cost (reverse)	0.22	0.08	0.49**
Outer setting	Student needs and resources	0.26*	−0.04	0.19
	Cosmopolitanism	0.2	0.26*	0.05
	Peer pressure	0.09	−0.03	0.08
	External policy and incentives	0.05	0.22	−0.25
Inner setting	Structural characteristics	0.14	0.22	−0.02
	Networks communications	0.03	0.17	−0.28*
	Culture	0.39**	0.48**	0.27*
	Tension for change	−0.33**	−0.16	−0.27*
	Compatibility	−0.1	0.02	−0.06
	Relative priority	0.31**	0.34**	0.12
	Organizational incentives and rewards	0.31**	0.18	0.01
	Goals and feedback	0.05	0	−0.24
Readiness for implementation	Learning climate	0.11	0.31**	0.34**
	Readiness for implementation	0.06	0.21	−0.03
	Leadership engagement	0.31**	0.44**	0.42**
	Available resources	0.28*	0.32**	0.01
	Access to knowledge and information	−0.19	−0.2	−0.133
Individual characteristics	Knowledge and beliefs about intervention	0.01	0.18	0.21
	Self-efficacy	0.05	0.33**	−0.01
	Individual stage of change	−0.02	0.16	0.47**
	Individual identification with organization	0.35**	0.13	−0.07
	Other personal attributes	0.08	0.33**	−0.14
Implementation process	Planning	0.18	0.50**	0.27*
	Engaging	0.23	0.54**	0.2
	Opinion leader	0.05	0.59**	0.19
	Implementation leaders (SWT)	0.033	0.2	0.37**
	Champions	0.21	0.42**	0.32**
	External change agents	0.08	0.31*	−0.19
	Key stakeholders	−0.06	0.15	−0.06
	Innovation participants	0.2	0.44**	0.11
	Executing	0.38**	0.40**	0.02
	Reflecting and evaluating	0.12	0.33**	0.39**

NA, not applicable due to lack of data to run correlations; *, p < 0.1; **, p < 0.05.

### Aim 3: Differences in Outcomes and Determinants Among New and Experienced Schools, and Influential Factors to Sustainment of SWITCH

Cross-case analyses facilitated understanding of distinguishing implementation determinants between inexperienced and experienced schools. For inexperienced schools, the highest ranked positive determinant was Leadership Engagement (Inner Setting-Readiness for Implementation), suggesting that school administration support was an important contributing factor. For experienced schools, the highest ranked positive determinant was Engaging-Innovation Participants (Implementation Process), indicating that student involvement and advocacy was helpful for success. Table 3 highlights the differences in mean scores between the two samples and distinguishing factors according to independent samples *t*-tests and large differences in means not detected through inferential testing. The two constructs which were statistically different were Readiness for Implementation (Inner Setting) and Self-efficacy (Characteristics of Individuals); experienced schools had positive and significantly higher means in these constructs indicating they were positive determinants to implementation. Other distinguishing constructs which had large score differences were Cosmopolitanism (Outer Setting), Peer Pressure (Outer Setting), Compatibility (Inner Setting), Engaging (Implementation Process), and Innovation Participants (Implementation Process). Experienced schools had higher means except for Peer Pressure, which was higher for inexperienced schools.

Table 5 highlights the distinguishing constructs which separated the two samples based on deductive coding, with salient interview extracts from school participants. Extracts were chosen to represent some of the diverging quotations from the two distinct subsamples and reflect the ways in which they experienced implementation facilitators and barriers according to each CFIR construct. An example from the Readiness for Implementation highlights a difference for inexperienced schools, “From the first meeting it sounded like it was just maybe teaching a couple of lessons, and [team member] was going to be doing most of it, but we quickly found out that, that really wasn't the case” and experienced schools, “I think our core team does really well at keeping these things planned, and sticking together, and letting administration know what we're doing, and getting the okay.” Another example from the Engaging construct highlights one perspective: “I don't feel we communicated well-enough to allow, or to educate the teachers on the importance of this program” (inexperienced). This is contrasted with an experienced school team member who said, “We had more success getting kids connected to their parents this year, compared to last year.” These differences highlight nuanced barriers/facilitators among the two samples.

**Table 5:** Distinguishing constructs between inexperienced and experienced schools.

**Construct**	**Inexperienced**	**Experienced**
Readiness for implementation**	“From the first meeting it sounded like it was just maybe teaching a couple of lessons, and [team member] was going to be doing most of it, but we quickly found out that, that really wasn't the case, but it worked out well, though. After we got over that initial shock of, ‘oh my gosh, it's a lot more work' but it did go well”	“I think our core team does really well at keeping these things planned, and sticking together, and letting administration know what we're doing, and getting the okay. But then going about and implementing it and getting the help we need to go it from the parents' community, just doing it that way”
Self-efficacy**	“It was mostly just me and [other teacher]. They were on board, but yeah, again, just, it was brand new to us, so we didn't know how to incorporate everyone else into it just fully yet”	“I had 100% confidence in my teachers, because we sat down the year before and chose to do it again. Like I said, I feel that they did the best that they could with the amount of time that they had to be able to implement additional curriculum into their already busy curriculum”
Cosmopolitanism	“I didn't do a good job of reaching out to the community to see if there was anyone interested in helping us”	“We did the Iowa Farm-to-School local food day this past school year. And we were able to get apples and cider from [local orchard], and then we got fresh leaf lettuce and vegetables from our own greenhouse here. And so, we were able to explain that to the kids, and [4-H officer] actually came in and helped during that”
Peer pressure	“So knowing kind of the ins and outs and how [SWITCH] should look from another previous school that had success with it, really helped us just kind of get going and get it running at our school”	“We used to put a lot of things of what our school did to share our ideas, and we didn't [in the community of practice] but we did on our school Facebook page and shared a lot in that way. So, this year I didn't feel like I knew what a lot of schools had done”
Compatibility	“Our biggest hurdle was finding time for sixth, seventh, and eighth grade classroom activities just because our schedule just didn't work out very well. We ended up having all six, seventh and eighth graders on Mondays for Switch. We're a really tiny school, but that's still about 50 kids, which is a large group in a gym trying to teach”	“I guess I just keep going back to our kickoff that and with the teachers came up with on that and how it directly coincided with SWITCH and they were phenomenal. I think that they had the opportunity to do it something within their classroom I think they would do it”
Engaging	“I don't feel we communicated well enough to allow, or to educate the teachers on the importance of this program. In that regard, I need to do a better job next year along with whoever's helping in this”	“We had more success getting kids connected to their parents this year, compared to last year. We only had two connected last year, and I was one of them. And I think we ended up with about 25 parents connected to kids, which doesn't sound like a lot… that's still something that we want to improve upon so they know what the kids are doing so they can then support it at home”

Orange, distinctively lower score; Green, distinctively higher score; ** Significantly different.

Finally, results from inductive coding with regard to sustainability revealed three overarching themes: (1) The importance of student awareness; (2) Keeping it simple; and (3) Integrating within school culture. Additional File 9 shows salient quotes from interviews related to these themes, with quotes separated by experience level. For the first theme, when wellness team members were asked if they felt their changes were sustainable, many pointed to the impact SWITCH has had on students as a key reason why the program would be maintained in their setting. One inexperienced school member said, “the kids have now become aware of [how] they can change what they do, view, and chew… And maybe next year when they see us in the hallway, it'll click and [they will] remember that kind of stuff.”

Many school wellness team members emphasized that while they could not implement all parts of SWITCH as much as they wanted to, they mentioned specific practices that seemed simple and granular which could be sustained. For example, one experienced school member said, “We've tried to do one thing at a time, to see if it was going to work. Changing the milk, we can do that. We do that all the time, now. And the brain breaks in the classroom, that's sustainable.” This indicates that the wellness teams are thinking more about the discrete practices/policies they have in place as opposed to the comprehensive nature of the program, which may be too overwhelming. Finally, participants discussed how they “really see this as it's just part of our culture” (inexperienced school) when discussing this question. One experienced school member explicitly discussed how they are planning to keep SWITCH going despite common challenges of staff turnover which are pervasive in schools:

I think everything we did is only going to be able to be built on. We've documented everything we did so if anything happened to any of us it's ready to go for the next group of people. And our district has worked really hard over the last couple of years with trauma informed care and social/emotional learning, so SWITCH ties into that with activity breaks, things like that. I foresee that that's just become common practice for our teachers.

This quote emphasizes the work that wellness teams have carried out to fully embed SWITCH within their systems so that it is compatible for their schools.

## Discussion

The aims of this study were to assess implementation outcomes of adoption, fidelity, and penetration of SWITCH to identify the factors that may influence sustainability. Grounded by CFIR, we discerned implementation determinants through a deductive approach and specifically examined the differences in outcomes and determinants among new and experienced schools. The use of CFIR as a guiding framework is novel in the school wellness setting, specifically the use of the framework systematic data analysis procedures, which facilitated a deep contextual understanding of relationships between implementation determinants and outcomes. Thus, a key innovation is the adaptation of a framework predominantly intended for healthcare settings (i.e., CFIR) to the school setting, marking an important advancement in the field of implementation science.

### Aim 1: Assess Implementation Outcomes

The SWITCH program represents a capacity-building process which allows school wellness teams to develop and sustain comprehensive programs of their own which in turn are more sustainable over time. The moderate-high rates of Penetration also correspond with self-reported Adoption of program best practices across the school setting. Implementation data from adoption, fidelity, and penetration measures highlight the differences between experienced and experienced schools, a result that aligns with preliminary findings from prior evaluations (13). However, the finding that all schools struggled to engage parents despite increased efforts in the 2020 academic year reflects a wealth of prior research documenting this lack of engagement problem (8, 50, 51). Outreach practices of sending communications (emails/newsletters) and holding events for parental engagement were the most frequently reported, reflecting similar trends with school nutrition program promotion (52). Such findings stress the need to view implementation outcomes as incrementally changing constructs that must be studied over time. This finding is consistent with generalized recommendations for continuous quality improvement models (37, 53).

### Aim 2: Assess Determinants of Implementation

The finding that Cosmopolitanism was higher in experienced schools, but Peer Pressure was lower than inexperienced schools, provides valuable information for how to support implementation efforts. Having links to other schools and organizations was viewed as a positive determinant of fidelity; interview data yielded some reasons for this, such as implementation support for delivering lessons and additional program materials and equipment, which may have further pushed a culture of health in school buildings. Although some initial research has demonstrated the positive role of external networks and support (54, 55), very little is known about the effectiveness of implementation strategies which provide targeted support from this domain. Accordingly, a potential implementation strategy for future work with schools may be to provide a local network of support, bringing together other sectors such as food retail and community centers, ultimately enhancing the culture of health in the community (56, 57).

### Aim 3: Differences Between New and Experienced Schools

For inexperienced schools, Leadership Engagement was the highest rated positive determinant of implementation. This is noteworthy since lack of support or involvement from school administration is a frequently reported barrier in school-based interventions (58–61). In SWITCH, administrators were able to be a part of the wellness team and attend conferences and trainings which likely enhanced their exposure to—and awareness of—school wellness programming. For all schools, Available Resources was the most negatively ranked determinant, indicating this was the biggest challenge for implementation. Examples from interviews highlighted the role of personnel time, equipment availability, and funding as supports for implementation. Therefore, an implementation strategy for inexperienced schools may be a cost-matching initiative through local county 4-H extension or through collaborating with community stakeholders, as described above and recommended through findings of Waltz et al. (62). County extension offices have been encouraged to support SWITCH programming in their county, so this finding supports the importance of this practice. Engagement of Extension in this way also enhances cross-sector collaborations to build more sustainable school and community health programming (57).

As expected, the Readiness for Implementation domain and findings from the SRWA assessment highlight the importance of capacity-building programs for systems change (9). Both Readiness for Implementation and Self-Efficacy were significantly higher for experienced schools than inexperienced, bolstering findings from the SWRA. This is not surprising, as items from the SWRA relate to Self-Efficacy in the individual and organizational psychological domains, such as “staff members at all levels share a belief that they can implement school wellness programs effectively.” Thus, implementation strategies to bolster capacity for implementation may be most appropriate. Within the D&I literature, the Expert Recommendations for Implementing Change (ERIC) research provides groundwork for selecting implementation strategies based on reported implementation challenges through models such as CFIR, facilitating tailored implementation support (62–64). For example, a CFIR-ERIC matching protocol conducted by Waltz et al. (62) and adapted by Cook et al. for school settings (65) highlighted that for Readiness for Implementation barriers, experts recommended “Assess for readiness and identify barriers and facilitators” as potential implementation strategies. In SWITCH, a core wellness team of at least three school staff members are trained over the course of a semester and complete the SWRA tool and School Wellness Environment Profile assessment, thus these strategies are already key components of the intervention model. Input from school stakeholders is often absent from the literature on implementation strategies, and a next step may be to include them in mapping procedures to advance the field.

The Implementation Process domain revealed that experienced schools ranked Engaging-general and Engaging-Innovation Participants distinctively higher than inexperienced schools, indicating these were more positively related to implementation. Related to innovation participants, youth advocacy in school wellness and health promotion has been demonstrated as an effective strategy for implementation and student health outcomes (66–68) and some studies are emerging regarding how student advocacy groups can be studied through a D&I lens (69). Engaging – Key Stakeholders was seen as a negative implementation determinant for all schools. Parents have been reported as the most difficult stakeholder group to engage in school-wide initiatives, and in previous cycles of SWITCH (13, 32, 50); however, some schools reported that when they did hold an event at school or at another academic-related event (i.e., parent-teacher conference), parents showed great interest. Thus, more research is needed to identify effective ways for engaging parents in school wellness, ideally with parents as the primary participants, to identify potential implementation strategies.

Finally, the inductive coding pertaining to sustainability revealed three primary themes which illustrate the strategies schools sought to maintain elements of SWITCH. A recent review highlights that most articles reporting facilitators/barriers to sustainment of interventions in schools cite factors from the Inner Setting as key determinants (9). Findings from the current study provide potential strategies that could be applied to mitigate barriers to sustainability, specifically (1) promoting student awareness and engagement, (2) focusing on a small number of key elements, and (3) integrating programming within school culture. These strategies were mentioned by participants as next steps for their wellness environment as formal implementation concluded, and all relate to potential barriers within the Inner Setting domain. However, it must be acknowledged that we were not able to test formalized strategies to enhance sustainability. Thus, a logical next step in this area may be to operationalize “sustainment” and to test the relative effectiveness of different strategies to enhance the sustainability of capacity-building interventions such as SWITCH. The present study provides insights into this development by identifying barriers and facilitators of adoption, fidelity and penetration.

## Limitations

There are some limitations that could influence interpretations from this type of evaluation. First, and most important, the COVID-19 pandemic led to school closures which prevented completion of the 12-week implementation cycle. Thus, it is not clear whether the documented differences between inexperienced and experienced schools would have persisted or varied. Further, to prevent overburdening school staff, we refrained from collecting checkpoint survey data once schools closed and began remote learning, which may have limited understanding of fidelity and adoption within schools. Finally, we acknowledge potential limitations of applying CFIR constructs and coding methods non-healthcare settings. Our study was one of the first to employ CFIR in school settings using a fully integrated mixed methods procedure. Therefore, the CFIR constructs/methods and their applications to school and community-based settings may need to evolve over time as replication of these methods occur. Ongoing work with SWITCH has utilized these findings, but the results provide generalizable insights about factors that influence the scale up and sustainment of interventions in other community-based settings (70). The process and systematic approach to the use of CFIR in the analyses also provide a guide for other school-based researchers seeking to utilize D&I methods to evaluate programming.

## Conclusions

The present study highlighted various determinants that influenced implementation and sustainability of SWITCH. The study added novel insights which can be tested and applied in other studies in school and community settings. Specifically, we documented that inexperienced schools face greater challenges and need tailored support, findings which indirectly document the gains in capacity built through previous iterations of SWITCH. The mixed methods approach used in the study was particularly important in understanding the factors influencing implementation and the greater challenges faced by inexperienced schools.

An advantage of CFIR in the project is that it provides a systematic method for enhancing the rigor and quality of implementation evaluations. Replication of the methods in other school-based projects would enable more effective comparisons. The adoption of “common measures” for implementation determinants and outcomes is already evident in other lines of research (70–73). Similar standardization efforts in school-based research would enhance generalizability and transferability of qualitative findings to other contexts and geographic locations. It is clear that what gets measured often is what gets achieved. By standardizing methods and measures, there is greater potential for enhancing implementation and sustainability of school-based interventions through incremental evaluation.

## Data Availability Statement

The raw data supporting the conclusions of this article will be made available by the authors, without undue reservation.

## Ethics Statement

The studies involving human participants were reviewed and approved by the Institutional Review Board (#14–651) at Iowa State University. The patients/participants provided their written informed consent to participate in this study.

## Author Contributions

GM and GW led the mixed methods design and evaluation components. RS and GM led the qualitative analysis procedures. RS and LL facilitated survey and interview data collection procedures. GM analyzed survey data and developed measures for school capacity. RR and JL provided feedback on analysis and interpretation of qualitative data. All authors contributed to the development of the research study and provided ongoing feedback throughout the implementation evaluation process and read and approved the final manuscript.

## Funding

USDA NIFA grant: 2015–68001-23242. The USDA was not involved in the design of the study and collection, analysis, and interpretation of data or writing of the manuscript.

## Conflict of Interest

The authors declare that the research was conducted in the absence of any commercial or financial relationships that could be construed as a potential conflict of interest.

## Publisher's Note

All claims expressed in this article are solely those of the authors and do not necessarily represent those of their affiliated organizations, or those of the publisher, the editors and the reviewers. Any product that may be evaluated in this article, or claim that may be made by its manufacturer, is not guaranteed or endorsed by the publisher.
