# Supplementary material for: Evaluation of a Large-Scale School Wellness Intervention Through the Consolidated Framework for Implementation Research (CFIR): Implications for Dissemination and Sustainability
Source: Front Health Serv. 2022 Apr 28;2:881639. doi: 10.3389/frhs.2022.881639 (PMC10012642; doi:10.3389/frhs.2022.881639)
Supplement: Supplementary file 1 [file Data_Sheet_1.docx]

**Supplementary Files**

Contents

[Additional File 1: Training and Implementation timeline 2](#_Toc89251781)

[Additional File 2: School Wellness Readiness Assessment (SWRA) Tool 4](#_Toc89251782)

[Additional File 3: Qualitative Interview Guide 8](#_Toc89251783)

[Additional File 4: Qualitative Interview Coding Consensus Document 15](#_Toc89251784)

[Additional File 5: CFIR Rating Rules (from www.cfirguide.org) 28](#_Toc89251785)

[Additional File 6: CFIR Coding Memo (first construct populated with example data) 31](#_Toc89251786)

[Additional File 7: Adoption (mean, SD) of best practices across the school context 43](#_Toc89251787)

[Additional File 8: Qualitative extracts aligning with relationships from interview data for all schools 44](#_Toc89251788)

[Additional File 9: Standards for Reporting Implementation Studies: the StaRI checklist for completion 48](#_Toc89251789)

#
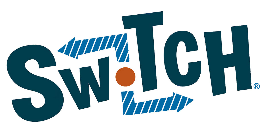
Additional File 1: Training and Implementation timeline

**Timeline 2019-2020**

**August 2019:**

- ISU Extension and Outreach provide orientation and materials for Youth Programming Specialists (YPS) and County Youth Coordinators (CYC) about 4H role in SWITCH (online and in person).
  - YPS/CYC come to Ames for 1 day Kick Off Training

**September 2019:**

- School register for SWITCH.
- SWITCH team schedules training webinars with school Core Teams

**October 2019:** *Orientation and Training for SWITCH*

- SWITCH Team hosts Introductory Webinar on SWITCH – 1 hour
- Schools and 4-H staff begin online module trainings.
- Schools receive guidance on initial steps and support from 4-H leaders (e.g. plan for kickoff / needs / roles)
- Core team and 4-H staff attend SWITCH School Wellness Conference in Ames (October 30 or 31)

**November 2019:** *Preparation for SWITCH Implementation*

- Core Team members determine regular meeting schedule to assess needs for school wellness
  (Regular meeting times are important since it creates structure and facilitates regular interactions – we recommend a weekly or every other week meeting plan)
- 4H partners (YPS/CYC) schedule meeting with Core Teams to facilitate planning
- 4H partners confirm presentation dates for school wide staff orientation/kick-off event planning.
- School Core Teams complete the School Wellness Environment Profile (SWEP) to assist in evaluating needs.
  - SWITCH Team prepares reports and return to schools upon completion).

**December 2019:** *Preparation for SWITCH Implementation continues*

- Core Teams attend Kickoff Webinar – 1 hour
- Core Teams enroll students into the SWITCH software
- Core Teams guide students to complete online Youth Activity Profile (YAP) to assess behaviors (Do, View, Chew)
- Core Teams plan promotional strategies and inform students / parents about SWITCH (generate buzz)
- Core Team and/or local 4H partner present SWITCH orientation to other school staff
- Implementation grant sent to schools upon completion of SWEP and YAP

**January 2020:** *Kickoff and SWITCH Implementation Begins*

- Schools finalize plan for SWITCH ‘Kick Off’ event and get parents and students excited about SWITCH
- Programming begins week of January 20^th^.

**February-March 2020:** *Programming continues*

- Core Teams promote school-wide engagement (use of posters / modules / website)
- Continue engagement with parents regarding SWITCH program
- Core Teams facilitate students use of web-based tracking for logging of Do, View, Chew behaviors

**April 2020:** *Wrap Up and Evaluation*

- Core teams participate in endpoint interviews
- Core Team members share successes on Community of Practice and complete program evaluation survey.
- 4-H Staff provide feedback and collect Group Enrollment Forms.

# Additional File 2: School Wellness Readiness Assessment (SWRA) Tool


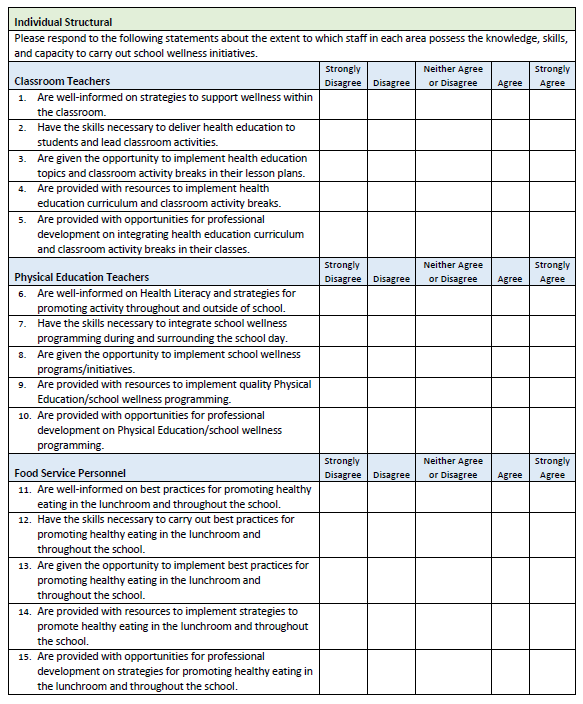


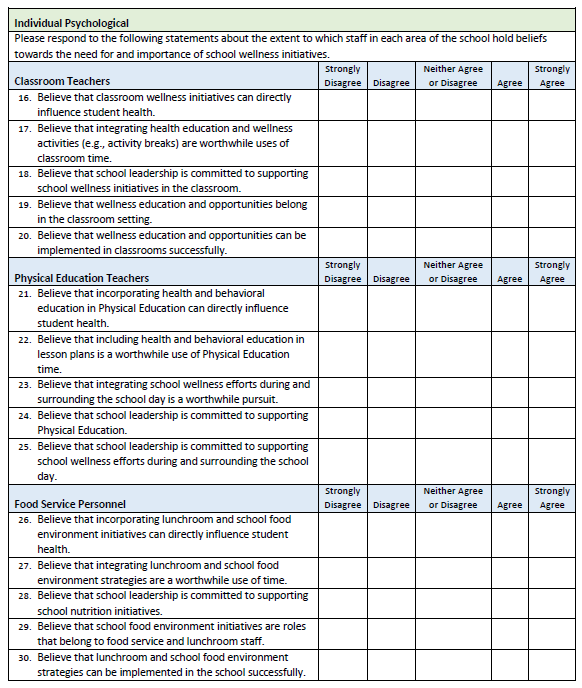


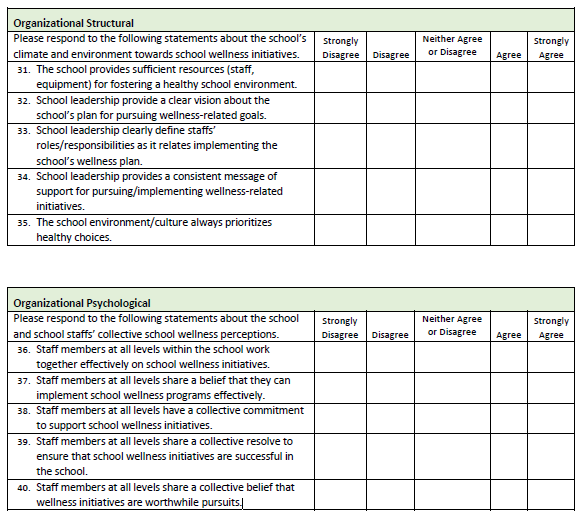


# Additional File 3: Qualitative Interview Guide

**Intervention Characteristics -** “First, I’d like to talk with you about some of the components of the SWITCH program and your perceptions toward different facets of implementation…”

1. Tell me about overall SWITCH implementation at your school.
   - What were your initial perceptions of the program?
   - Have these perceptions change at all over the course of SWITCH implementation?
     - How has the current COVID-19 pandemic and school closures affected your experiences?
   - What did your administration and other school leaders think of the program?
2. What kind of information or evidence were you aware of that made you believe this program would work in your school?
   - What had you read or heard (social media, other sources) that made you think SWITCH could work for your school?
   - How did this knowledge affect your perception of the intervention? Other staff?
3. What kinds of changes or alterations did you have to make to the intervention that it worked effectively in your setting?
   - What were some challenges you faced when adapting SWITCH to your school?
   - What were some examples of successes you experienced?
   - What adaptations did you have to make when schools stopped in-person instruction as a result of COVID-19?
4. How complicated did you find the SWITCH program?
   - What were the most difficult parts to implement? Why?
   - Can you provide some examples of these complicated aspects? How did you begin to tackle implementation?
5. What is your perception of the quality of the modules, posters, and other SWITCH materials that were provided?
   - How did your opinions impact how you used these materials?
6. What supports, such as online resources, marketing materials, or a toolkit, were available to help you implement and use the intervention? (provide examples here: YouTube site, social media, Canvas course, SWITCH website- ask how these were used)
   - How did you access these materials?
   - What strategies did you take to make the most use of these resources? Did you use some more than others?
   - Did things change during school closures? How did you modify these resources for students over the last four weeks of the program?
7. How did these available resources affect implementation in your setting?
8. How confident were you that you would be able to successfully implement SWITCH?
   - What gave you that level of confidence (or lack of confidence)?
9. How confident do you think your colleagues felt about implementing the intervention?
   - What gave them that level of confidence (or lack of confidence)?

**Outer Setting -** “Let’s talk a little about some of the school and external factors that might have played a role in implementation this year…”

1. To what extent do you feel administrators are aware of the needs and preferences of the students and staff at your school?
   - How "in touch" are the administration with health and wellness programs like SWITCH at your school?
2. How well do you think SWITCH meets the needs of the teachers and staff at your school?
   - What are the benefits you see when implementing SWITCH in your school?
   - In what ways do you think the SWITCH program meet their needs? E.g., student health, teacher morale, improved school culture?
3. How do you think teachers and staff responded overall to the program?
   - What barriers did individuals served by your organization face when participating in the program?
   - (if barriers listed) What could be done to better engage these staff and increase their involvement? Could this come from SWITCH or from the core team/within the school setting?
4. How would you describe the level of support you received from your county youth extension officer(s)?
   - To what degree did they facilitate SWITCH implementation?
   - If experienced school: Did this engagement differ from last year? If so/not- how?
   - How might this support system be improved for future iterations of SWITCH?
5. How would you describe the level of support you received from the SWITCH team at Iowa State?
   - How did you feel about the level of communication you received from the SWITCH team? To what degree were weekly emails beneficial to your core team’s success?
   - What aspects of communication/support were most beneficial to your school? What about when schools closed due to COVID-19?
   - How could the SWITCH team’s involvement be improved?
6. Can you tell me what you know about any other schools that have implemented SWITCH or other programs?
   - How has this information influenced the decision to implement SWITCH?
   - To what extent are other schools implementing the program?
7. As you might know, the United States Department of Agriculture (USDA) and the United States government instituted the Final Rule in 2016 which mandated that schools must evaluate their wellness polices every three years to promote continuous development. How do you think this impacted your school wellness environment and/or perceptions of SWITCH?
   - How does SWITCH affect your school’s ability to meet these guidelines?

**Inner Setting** - “These next questions will ask a bit more about your school and your work environment in relation to SWITCH.”

1. Can you describe your working relationship with administration?
   - Your supervisor? Supervisors of other colleagues?
2. How would you describe the culture of your school building?
   - Do you feel like the culture of your own department/grade level/subject area is different from the overall organization? In what ways?
3. How do you think your school culture (general beliefs, values, assumptions that people embrace) affected the implementation of SWITCH programming?
   - Can you describe an example that highlights this?
4. How do people feel about the activities and processes related to the SWITCH program? For example, using the online platform, facilitating tracking, making changes in the lunchroom, physical education, and other school settings?
   - To what extent does SWITCH fill a need in your school and the other stakeholders?
   - How well does SWITCH fit with your values and norms and the values and norms within your school?
5. To what extent did SWITCH and overall wellness take a backseat to other high-priority initiatives going on now?
   - How important do you think it is to implement the intervention compared to other priorities?
   - How important is it to others, such as your coworkers or leaders, to implement the intervention compared to the other priorities?

**Readiness for Implementation**

1. What level of endorsement or support have you seen or heard from school and/or district administration for implementing SWITCH?
2. What level of involvement has the administration at your school had so far with the intervention?
   - Did they know about how SWITCH was being implemented?
   - What kind of support have they given you? Can you provide specific examples?
3. How did you perceive the utility of the training received prior to program implementation?
   - What about the webinars (pre-conference (October 2019 and post-conference, December 2019)?
   - What about the open enrollment webinars that were offered in spring during implementation?
   - What about the SWITCH school wellness conference (2019)?
4. What would you say was the most beneficial aspect of training for your school?
   - Why was this? How did it help your core team succeed in SWITCH?
   - If returning- what was most beneficial?
5. What suggestions would you have to improve the training program?
6. What were some of the steps you took to plan for implementation?
   - What role has your plan for implementation played during implementation?
   - Was it used to compare planned with actual progress?
7. Who were the key influential individuals to get on board with this program?
   - What are these stakeholders saying about SWITCH? (this could have been other teachers, parents, students, county extension staff, and outside stakeholders who are influential in the school setting).
   - To what extent did they influence others' use of the program? The success of the implementation?
8. Who led implementation of the intervention? Was it mostly you or did you work as a Core Team
   - How did your group come into this role? Appointed? Volunteered? Voluntold?
   - What attributes or qualities do you feel made you the best leader(s) of this program? Does this person have sufficient authority to do what is necessary to implement the intervention?
9. Other than the Core Team or yourself, are there people in your school who went above and beyond what was expected of them, such as other staff, students, and external stakeholders?
   - Were they formally appointed in this position, or was it an informal role?
   - How do you think they helped with implementation? Getting other stakeholders on board?
10. Was there anyone outside the school building who helped to implement SWITCH? (i.e. outside organization, local business, authority)?
    - Please describe this person/group?
    - How did they get involved?
    - What was their role?
    - How did they influence implementation?
11. SWITCH is a comprehensive intervention which is implemented in all school settings but specifically core classrooms, the lunchroom, and physical education settings. Given this broad reach, what strategies did you use to encourage individuals to commit to using the SWITCH intervention and materials?
    - Which individuals did you target specifically?
    - How frequently and how did you communicate with them?
    - Did this change during school closures due to COVID-19?
12. What was your communication or education strategy for getting the word out about SWITCH to parents and other staff?
    - What materials/methods did you use? For example: e-bulletin boards, emails, presentations?
    - What process did you use to communicate? For example, going to staff meetings, talking to people informally?
13. What could the SWITCH team offer to help facilitate school-level buy-in in the future?
    - Can you provide some examples?
14. Parent engagement was cited as a challenge for many schools in 2019. What strategies did you use to engage parents in programming this year?
    - Email
    - Social media
    - Family events
    - SWITCH website
    - In-person
15. What about after COVID-19?
16. What successes and challenges did you face when trying to engage parents?
    - Can you provide examples of these experiences?
    - Did you do anything differently when schools closed due to COVID-19? If so, what new/different strategies did you implement to engage parents?
17. How do you think parents would want to be engaged in programming? Did they provide any feedback to you about their involvement with SWITCH?
    - How do you think their engagement changed (if at all) during school closures/COVID-19?
18. What strategies or supports do you feel would be helpful to better engage parents in SWITCH and other wellness programming?

**Implementation Process – “**These final questions will ask you about the implementation process at your school and plans for sustainability in the future.” Five main areas: posters, modules, student online tracking, parent engagement, student leadership

1. Do you feel the SWITCH program was implemented according to the best practices?
   - [If Yes] Can you describe this?
   - [If No] Why not?
   - Did this change at all during school closures due to COVID-19? If so/not, why do you think this would be?
2. How well do you feel SWITCH was integrated across the school setting? What were some ways that you were able to reach students in your school through programming?
   - Did you have challenges with these processes?
3. How often were you able to use modules, posters, and encourage student tracking in each setting? (i.e., once a week/ multiple times per week/ every day?)
   - Classrooms/homerooms, Physical education, Lunchroom?
4. How did you engage students as leaders in SWITCH? What did this process look like? What specific tasks did your students take on?
   - To what degree did they facilitate/impact implementation?
   - What were some successful moments you had in engaging students?
   - Did you face barriers? How did you overcome them?
5. What successes have you experienced with the program? Tell me about these and how these successes were achieved (optional)
6. What has been difficult or challenging for you during SWITCH implementation?
   - Were these challenges environmental (i.e. schedule, school building) or interpersonal (difficulty getting buy-in from others, lack of interest from teachers, etc.)?
   - (if minimal answers were provided) Can you provide an example of this? How did you overcome this challenge?
7. Let’s talk about the goals for SWITCH that you set back at the beginning (mention goals here). What progress have you made toward these goals?
   - Were these goals realistic or too challenging?
   - What successes and challenges have you experienced in putting these goals into action?
   - How competent did you feel in implementing these goals?
   - What kind of support did you have with implementing these goals?
8. Let’s talk about the school wellness environment. What changes, if any, have you seen in your school environment or culture? (were there any big wins that you have had?)
   - What are the main areas you identified as priorities from the SWEP report (include priority areas here)?
   - Can you tell me what steps have been taken to improve these areas?
   - How do you think the school environment will facilitate/impede these changes?
9. How were you able to monitor implementation of SWITCH best practices over the time schools were in session?
   - How did this help you stay on track during the implementation phase?
   - What best practices/strategies did you find most challenging to implement during COVID-19 school closures?
10. Think of the changes you have made in your school setting. To what degree do you think these changes are sustainable?
11. What do you think the next steps are for improving your school wellness environment?
    - What goals can you set for the next few months?
    - How are you going to achieve these goals?
12. What about your school wellness policy? How do you think the work you have done in SWITCH can relate to your wellness policy?
13. The goal of the ISU SWITCH team is to disseminate the program so that all schools across the state have the opportunity to engage in this initiative. What would you tell teachers at other schools if they asked you about implementing SWITCH at their school?
14. If your school had the opportunity to do SWITCH again this year, is this something you would be interested in?
15. On a scale from 1-5 (1 is not at all; 5 is very), how likely are you to recommend SWITCH to another school?
16. On a scale from 1-5 (1 is not at all; 5 is very), how beneficial was SWITCH for your school wellness environment?
17. On a scale from 1-5 (1 is not at all; 5 is very), how engaged and supportive was your county extension staff member?
18. What other things do you want to ask us or do you want to tell us?

# Additional File 4: Qualitative Interview Coding Consensus Document

**SWITCH 2020 Coding Consensus**

Guiding Framework: Consolidated Framework for Implementation Research

| **Facility** | **Coding Example/Source** | **Potential Misconceptions** |
| --- | --- | --- |
| **I. Innovation Characteristics** |  |  |
| A. Innovation Source | - Did they know that it was an Iowa State program?  - Did they know it was validated? | - School stakeholders’ perceptions of the program (potential for success) – suggest coding under “readiness for implementation-leadership engagement” or “Engaging- opinion leaders” |
| B. Evidence Strength & Quality | Perceptions about SWITCH and beliefs that it would work (before implementation)  Heard from other schools that SWITCH would work (also peer pressure). | Knowledge of Iowa State and where the program comes from- code in innovation source |
| C. Relative Advantage | When schools talk about prior programming they have implemented and compared it to SWITCH/talked about why SWITCH was better/worse | Statements that imply the need for change or dissatisfaction with current school climate (e.g., “our students sit too much”)- instead code under tension for change or patient needs/resources |
| D. Adaptability | Statements regarding how materials were adapted (i.e., modules/resources) or broadened the audience for programming (i.e., moving to K-12) | “it easily fit in our curriculum”- code instead to compatibility |
| E. Trialability | Reference to only working with one/fewer classroom(s) in the prior year and then full rollout this year (evaluate if they felt it was a good/bad thing) | Exclude other data that does not relate to their own testing/pilot work in their school |
| F. Complexity | Examples of how schools initially might have struggled but once they developed a plan they were able to implement. | Difficulty using modules due to design/clutter/lack of clarity regarding how to access information (i.e., website, canvas course, social media, etc.) – instead code to design quality and packaging  Difficulty with getting team members together- code instead to engaging- formally appointed implementation leaders |
| G. Design Quality & Packaging | Issues related to clarity of information, quality of resources, and communication strategies. Parent newsletters, social media posts/content. | Reference to program materials as being too difficult to use/too complex but not a specific reason or barrier- suggests low motivation and planning; code in either “other personal attributes” or “planning”. Exclude statements regarding the presence or absence of materials and code to Available Resources. |
| H. Cost | Extracts related to cost of running SWITCH (e.g., if they said they used grant funds to buy vegetables which are needed in future for ongoing programming). | If schools stated that they didn’t have enough funding to implement the program, code under “available resources” |
| **II. Outer Setting** |  |  |
| A. Needs & Resources of Those Served by the Organization | Statements that demonstrate school awareness of their student needs, such as “our students just don’t move enough” or “they only have X PE lessons per week/recess periods per day” – these illustrate that schools are adopting SWITCH because they want to impact their students’ health and wellness | Exclude statements that do or do not demonstrate a strong need for the innovation and/or that the current situation is untenable, e.g., statements that SWITCH is necessary or not at all needed, and code to Tension for Change. Don’t code how schools are involving students in programming- instead code to engaging-innovation participants |
| B. Cosmopolitanism | Statements from schools that talk about their involvement with local and state organizations (i.e., IAHPERD, other teacher organizations), linked to their county extension system, ability to go to conferences,.  Also- data on their programs and networks with other schools and organizations. | Any school-specific stakeholder engagement (i.e., parents)- code instead to networks and communications and engaging- opinion leaders |
| C. Peer Pressure | Statements from schools related to their knowledge about what other schools have done (i.e., from the conference, nearby schools). Negative instances could be coded as being “voluntold” to undertake SWITCH by a principal/more senior teacher. | Statements not pertaining to influential aspects. If the school found out about SWITCH on their own, data shouldn’t be coded here but potentially to evidence strength. |
| D. External Policy & Incentives | When schools talk about the final rule, local wellness policies, or other mandates which would influence participation in SWITCH. Also, if they report that their job performance somehow includes leading initiatives like SWITCH. |  |
| **III. Inner Setting** |  |  |
| A. Structural Characteristics | Social structure- statements that refer to how the core team (or leader) is peripheral or distal to the rest of their school, i.e., if they are in an isolated unit or if the school structure is unified. If decision making is centralized (i.e., “top-down approach”) |  |
| B. Networks & Communications | Statements that reflect the degree to which collaboration with different sections of the school setting took place in SWITCH implementation. Negative examples might be a lack of communication/synergy with lunchroom, PE, classroom, etc. | Don’t code “lack of buy-in” from other staff, and instead code these to engaging key stakeholders. |
| C. Culture | When asked about their school culture, include statements about the degree to which the culture is a collaborative one or if there seems to be a culture of wellness in the school. Or, statements about the ability to “get things done” in the school setting. Negative examples could relate to how slowly things tend to move in school settings and a lack of clarity/opportunity for innovation. |  |
| D. Implementation Climate |  |  |
| 1. Tension for Change | Need for change is described in the interview, through the “need to increase the wellness environment” and reasons why SWITCH is needed in the school context. | Exclude comments linked to needs of individuals (i.e., students need movement to enhance learning etc.) |
| 2. Compatibility | Statements that indicate the intervention didn’t need to be adapted and aligned with the procedures/operations already in existence | Statements about the degree to which SWITCH was a priority for schools- code to relative priority |
| 3. Relative Priority | The degree to which SWITCH was viewed as a priority in school operations, or “took a back seat” in terms of programming due to other pressures such as testing, core content, etc. | If SWITCH was not prioritized because it was not viewed as being compatible, code under compatibility. |
| 4. Organizational Incentives & Rewards | Statements related to internal incentives or rewards for SWITCH core teams. Talk about lack of incentives also – as these could be interpreted as negative influences or capacity |  |
| 5. Goals & Feedback | Example: If school core teams report their desires to enhance wellness in the whole building and talk about how SWITCH helps them to address these goals. | Exclude core team meetings to plan implementation as part of the formal SWITCH program, as this likely ends when the 12-week phase does. |
| 6. Learning Climate | Statements which emphasize the notion of “learning together” and that the school core team/leadership acknowledges the struggles with trying something new. Negative examples include where core teams are worried about failing as it would not be taken well/addressed as a learning step |  |
| E. Readiness for Implementation | General readiness for implementation (i.e., not using specific examples but expressing confidence for implementation) | Nothing too specific- these would go in the sections below |
| 1. Leadership Engagement | Mention of principals/superintendents or curriculum directors showing support for SWITCH and becoming engaged in some way | Exclude statements showing direct implementation of SWITCH- i.e., leading lessons or activities. Code these under engaging- champions. |
| 2. Available Resources | Money, time, and space allocations to SWITCH. This can be positive and negative- and there may be examples of both (i.e., not enough time but there is space etc.) -this might be a mixed coding example | Exclude comments about training and instead code to access to knowledge -below |
| 3. Access to Knowledge & Information | Experiences and perceptions of training (i.e., fall conference, webinars, etc.), and SWITCH program materials. A negative example could relate to core teams’ usage of various sources to access the right information. From the website: “They sent us to training about 9 months before implementation so by the time things were finally ready, I forgot what to do.”- this could relate to the gap between the conference and the actual implementation phase.  Also, reference to programmatic support (logistics and resources) from the ISU SWITCH team should be coded here. | Exclude findings relating to networking within the school and trying to engage key stakeholders. |
| **IV. Characteristics of Individuals** |  |  |
| A. Knowledge & Beliefs about the Innovation | Positive or negative beliefs about the intervention. This could include data that was coded to “evidence strength” but would not be limited to perceptions of pre-implementation |  |
| B. Self-Efficacy | Perceived ability (either within one individual or within the collective core team) regarding implementation. Negative examples could address lack of self or collective efficacy to implement the intervention. |  |
| C. Individual Stage of Change | Statements pertaining to perceived need to change or perceived personal readiness for change/SWITCH. |  |
| D. Individual Identification with Organization | Degree to which core teams feel valued within school settings | Communications with others inside the school setting- code instead to networks in the inner settings |
| E. Other Personal Attributes | Here code statements/data related to motivation and experience with programs such as SWITCH. Do the core teams seem/feel motivated to implement motivation? Is there a mix of motivations within core team? |  |
| **V. Process** |  |  |
| A. Planning | Statements/data related to planning such as working together at the conference, meeting as a core team, and other planning activities. |  |
| B. Engaging | Overall engagement of key stakeholders (i.e., rest of the school, students, staff, parents) |  |
| 1. Opinion Leaders | Engaging staff in the intervention. From the website: “Individuals in an organization who have formal or informal influence on the attitudes and beliefs of their colleagues with respect to implementing the intervention” – so how did they engage other staff and even administration? Was this lacking? |  |
| 2. Formally Appointed Internal Implementation Leaders | What is the role of the core team in implementing SWITCH? What did each person do? Here is where we could tease out whether there is “shared” or “sole” leadership- i.e. how much collaboration existed within the core team and how much was it a one-person effort? | Any data linked to motivation of the core team goes in “personal attributes” |
| 3. Champions | Here we code data in relation to how an individual/group outside the core team stepped up, and potentially any others that “stepped up” to the plate in terms of leading implementation. This could be students, teachers, admin, or a parent. |  |
| 4. External Change Agents | Extracts in relation to how **extension** staff were involved with implementation, through peripheral support or direct efforts within the school setting. |  |
| 5. Key Stakeholders | This isn’t in the online version, but I think we might be wise to include **parents** and other community stakeholders here. |  |
| 6. Innovation Participants | Engaging **students**- statements on the strategies used to recruit youth ambassadors and/or engage them in programming. Statements on their involvement coded here. Examples such as “our students loved the posters and kept asking for the answer to the trivia questions”, or “they keep asking to do taste tests” | If students took initiative and led a program or activity, and showed leadership, then they would go in champions. |
| C. Executing | Statements and data here that speak to how SWITCH quality elements were used and to address areas of strong/weak implementation in relation to quality elements and best practices (i.e., modules, tracking, posters, weekly meetings, etc.) | Information about factors within the school setting (i.e., involvement of people) that may impact implementation. |
| D. Reflecting & Evaluating | Extracts/statements which highlight the degree to which core teams were able to evaluate ongoing implementation “in real time” aka: as it happened, and make changes accordingly. | Exclude data which speaks to the (lack of) relation of SWITCH to ongoing goals and plans of the school- code to compatibility instead. Also, retrospective reflection and appraisal can go in the Executing Phase. Or if they talk about lack of engagement and speak of how they can improve for next year, this would go in the appropriate Engaging subsection. |

| **Coding Valence and Strength** | **-2** | **-1** | **0** | **X** | **+1** | **+2** |
| --- | --- | --- | --- | --- | --- | --- |
|  | If school provided examples of how a specific facet hindered implementation or mentioned it repeatedly as a barrier | If mostly negative but some positive, try to code here to reflect the weight of the data. | General comments that relate to this construct but it’s difficult to tell if there was a positive or negative impact. | When there is discordance between two (or more) people on the same interview, and their points of view are about the same construct. Unless they come to an agreement or one’s point is addressing a more minor component. | If mostly positive but some negative, try to code here to reflect the weight of the data. i.e., “mostly positive” but some neutral or slightly negative. | If schools provided a concrete example of how this (construct) impacted implementation, or if they repeatedly mentioned how this positively influenced their implementation. |

# Additional File 5: CFIR Rating Rules (from [www.cfirguide.org](http://www.cfirguide.org))


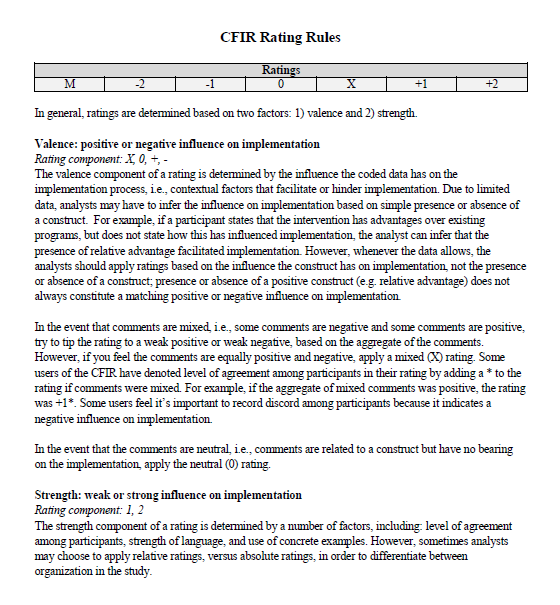


# Additional File 6: CFIR Coding Memo (first construct populated with example data)

**Organization Memo Report**

**Analyst(s): GMM & RS**

**Organization: School 1000**

**Interview Participants:**

**CFIR Constructs:**

**I. INNOVATION CHARACTERISTICS**

**A Innovation Source**

*RATING: OVERALL -2 (ANALYST ONE -2, ANALYST TWO -2)*

*SUMMARY:* The core team didn’t believe that SWITCH was a reliable or beneficial program and struggled to implement it.

*RATIONALE:* The program was developed nationally and the organization did not have a choice whether or not they would implement the program. They mentioned that their principal approached them and asked them to do this, so therefore felt rather “voluntold”.

*DATA:*

“Honestly, I feel like our admin just said “hey we’re doing this program and I’m asking you to lead it” kinda thing, so I feel like we never really had a say in it. I don’t know if I would have chosen SWITCH to be honest”

**B Evidence Strength & Qualit**y

*RATING: OVERALL ___ (ANALYST ONE ___, ANALYST TWO ___)*

*SUMMARY:*

*RATIONALE:*

*DATA:*

**C Relative Advantage**

*RATING: OVERALL ___ (ANALYST ONE ___, ANALYST TWO ___)*

*SUMMARY:*

*RATIONALE:*

*DATA:*

**D Adaptability**

*RATING: OVERALL ___ (ANALYST ONE ___, ANALYST TWO ___)*

*SUMMARY:*

*RATIONALE:*

*DATA:*

**E Trialability**

*RATING: OVERALL ___ (ANALYST ONE ___, ANALYST TWO ___)*

*SUMMARY:*

*RATIONALE:*

*DATA:*

**F Complexity** (Reverse rated, low complexity understood as positive)

*RATING: OVERALL ___ (ANALYST ONE ___, ANALYST TWO ___)*

*SUMMARY:*

*RATIONALE:*

*DATA:*

**G Design Quality & Packaging**

*RATING: OVERALL ___ (ANALYST ONE ___, ANALYST TWO ___)*

*SUMMARY:*

*RATIONALE:*

*DATA:*

**H Cost**

*RATING: OVERALL ___ (ANALYST ONE ___, ANALYST TWO ___)*

*SUMMARY:*

*RATIONALE:*

*DATA:*

**II. OUTER SETTING**

**A Needs & Resources of Those Served by the Organization**

*RATING: OVERALL ___ (ANALYST ONE ___, ANALYST TWO ___)*

*SUMMARY:*

*RATIONALE:*

*DATA:*

**B Cosmopolitanism**

*RATING: OVERALL ___ (ANALYST ONE ___, ANALYST TWO ___)*

*SUMMARY:*

*RATIONALE:*

*DATA:*

**C Peer Pressure**

*RATING: OVERALL ___ (ANALYST ONE ___, ANALYST TWO ___)*

*SUMMARY:*

*RATIONALE:*

*DATA:*

**D External Policy & Incentives**

*RATING: OVERALL ___ (ANALYST ONE ___, ANALYST TWO ___)*

*SUMMARY:*

*RATIONALE:*

*DATA:*

**III. INNER SETTING**

**A Structural Characteristics**

*RATING: OVERALL ___ (ANALYST ONE ___, ANALYST TWO ___)*

*SUMMARY:*

*RATIONALE:*

*DATA:*

**B Networks & Communications**

*RATING: OVERALL ___ (ANALYST ONE ___, ANALYST TWO ___)*

*SUMMARY:*

*RATIONALE:*

*DATA:*

**C Culture**

*RATING: OVERALL ___ (ANALYST ONE ___, ANALYST TWO ___)*

*SUMMARY:*

*RATIONALE:*

*DATA:*

**D Implementation Climate**

***1 Tension for Change***

*RATING: OVERALL ___ (ANALYST ONE ___, ANALYST TWO ___)*

*SUMMARY:*

*RATIONALE:*

*DATA:*

***2 Compatibility***

*RATING: OVERALL ___ (ANALYST ONE ___, ANALYST TWO ___)*

*SUMMARY:*

*RATIONALE:*

*DATA:*

***3 Relative Priority***

*RATING: OVERALL ___ (ANALYST ONE ___, ANALYST TWO ___)*

*SUMMARY:*

*RATIONALE:*

*DATA:*

***4 Organizational Incentives & Rewards***

*RATING: OVERALL ___ (ANALYST ONE ___, ANALYST TWO ___)*

*SUMMARY:*

*RATIONALE:*

*DATA:*

***5 Goals & Feedback***

*RATING: OVERALL ___ (ANALYST ONE ___, ANALYST TWO ___)*

*SUMMARY:*

*RATIONALE:*

*DATA:*

***6 Learning Climate***

*RATING: OVERALL ___ (ANALYST ONE ___, ANALYST TWO ___)*

*SUMMARY:*

*RATIONALE:*

*DATA:*

**E Readiness for Implementation**

***1 Leadership Engagement***

*RATING: OVERALL ___ (ANALYST ONE ___, ANALYST TWO ___)*

*SUMMARY:*

*RATIONALE:*

*DATA:*

***2 Available Resources***

*RATING: OVERALL ___ (ANALYST ONE ___, ANALYST TWO ___)*

*SUMMARY:*

*RATIONALE:*

*DATA:*

***3 Access to Knowledge & Information***

*RATING: OVERALL ___ (ANALYST ONE ___, ANALYST TWO ___)*

*SUMMARY:*

*RATIONALE:*

*DATA:*

**IV. CHARACTERISTICS OF INDIVIDUALS**

**A Knowledge & Beliefs about the Innovation**

*RATING: OVERALL ___ (ANALYST ONE ___, ANALYST TWO ___)*

*SUMMARY:*

*RATIONALE:*

*DATA:*

**B Self-Efficacy**

*RATING: OVERALL ___ (ANALYST ONE ___, ANALYST TWO ___)*

*SUMMARY:*

*RATIONALE:*

*DATA:*

**C Individual Stage of Change**

*RATING: OVERALL ___ (ANALYST ONE ___, ANALYST TWO ___)*

*SUMMARY:*

*RATIONALE:*

*DATA:*

**Individual Identification with Organization**

*RATING: OVERALL ___ (ANALYST ONE ___, ANALYST TWO ___)*

*SUMMARY:*

*RATIONALE:*

*DATA:*

**E Other Personal Attributes**

*RATING: OVERALL ___ (ANALYST ONE ___, ANALYST TWO ___)*

*SUMMARY:*

*RATIONALE:*

*DATA:*

**V. PROCESS**

**A Planning**

*RATING: OVERALL ___ (ANALYST ONE ___, ANALYST TWO ___)*

*SUMMARY:*

*RATIONALE:*

*DATA:*

**B Engaging**

*RATING: OVERALL ___ (ANALYST ONE ___, ANALYST TWO ___)*

*SUMMARY:*

*RATIONALE:*

*DATA:*

***1 Opinion Leaders***

*RATING: OVERALL ___ (ANALYST ONE ___, ANALYST TWO ___)*

*SUMMARY:*

*RATIONALE:*

*DATA:*

***2 Formally Appointed Internal Implementation Leaders***

*RATING: OVERALL ___ (ANALYST ONE ___, ANALYST TWO ___)*

*SUMMARY:*

*RATIONALE:*

*DATA:*

***3 Champions***

*RATING: OVERALL ___ (ANALYST ONE ___, ANALYST TWO ___)*

*SUMMARY:*

*RATIONALE:*

*DATA:*

***4 External Change Agents***

*RATING: OVERALL ___ (ANALYST ONE ___, ANALYST TWO ___)*

*SUMMARY:*

*RATIONALE:*

*DATA:*

***5 Key Stakeholders***

*RATING: OVERALL ___ (ANALYST ONE ___, ANALYST TWO ___)*

*SUMMARY:*

*RATIONALE:*

*DATA:*

***6 Innovation Participants***

*RATING: OVERALL ___ (ANALYST ONE ___, ANALYST TWO ___)*

*SUMMARY:*

*RATIONALE:*

*DATA:*

**C Executing**

*RATING: OVERALL ___ (ANALYST ONE ___, ANALYST TWO ___)*

*SUMMARY:*

*RATIONALE:*

*DATA:*

**D Reflecting & Evaluating**

*RATING: OVERALL ___ (ANALYST ONE ___, ANALYST TWO ___)*

*SUMMARY:*

*RATIONALE:*

*DATA:*

# Additional File 7: Adoption (mean, SD) of best practices across the school context

Note: Note: 0= not at all; 2= somewhat; 3= fully implemented across the school; modules = implementation of curricular modules; posters = promotional and interactive posters; tracking = helping students conduct behavioral self-monitoring for physical activity, nutrition, and screen time.

# Additional File 8: Qualitative extracts aligning with relationships from interview data for all schools

| **Domain** | **Construct** | **Relationship with outcome (+/-)** | **Interview Extract** |
| --- | --- | --- | --- |
| Intervention Characteristics | Innovation Source | Fidelity (+) | “I think it helps that it's not [wellness team] saying, ‘You guys should do this.’ We're bringing a program that's research based through the Extension office, so there's great buy in, knowing that this has been practiced before, it's been successful in [region], and we're not just asking them to hop on any old thing we found online. So I feel like they were totally on board.” |
|  | Complexity | Fidelity (+) | “At least for me, the elementary part was pretty straightforward. Middle school, kind of the same. At least for middle school, how to get everyone somewhat like, ‘Hey, how do we make them stay on track and just try and make sure we reach everyone?’ That's the most, I guess, complicated part of it.” |
|  | Cost | Adoption (+) | “It doesn't cost us any more to include everybody, than what it is. So that's a great benefit to the whole school that nobody's left out. Sometimes pre-k doesn't do it, but everybody's involved, which helps us tremendously.” |
| Outer Setting | Student Needs and Resources | Penetration (+) | “So [students] had so much fun and really the junior high teachers said, "I wish they could realize how much better behaved they were after a fun recess when they were moving around and giggling and letting off that stress by giggling and moving than when they just sit there and gossip." That's about all girls would do normally if you didn't have an activity going. So that I thought was great. We had more teachers involved to do all that. That was really kind of fun.” |
|  | Cosmopolitanism | Fidelity (+) | “We did a lot with 5-2-1-0 also this year [and] SWITCH because we became a 5-2-1-0 site, as well. And so, we did a lot of that with SWITCH at the same time, so that really made it good to just incorporate all that together.” |
| Inner Setting | Tension for Change | Penetration (-)  Adoption (-) | “Also, we always say... We had some teachers that are like, "Oh, I need to keep this kid for PE." Like, I don't think so, unless he's coming in some other time to do it. So I mean, I think they're starting to understand that the kids need to be in there, that they need that [active] time.” |
|  | Relative Priority | Fidelity (+)  Penetration (+) | “I don't know that I would say it really took a backseat. I don't know that there was other high priority issues. There were things that we had to do, yes, but I think it was just an equal like, ‘You got to do that. One way or another, we'll get done, and it's going to happen.’ ” |
|  | Culture | Fidelity (+) | “I think the supportive atmosphere helped, that everyone could get on board and see that this was important and how we could work together to do it and help each other out.” |
|  | Networks & Communications | Adoption (-) | “The PE teacher, I had no interaction with. So I guess, next year my goal would be just to get those teachers early in the fall to buy into it a little bit more.” |
| Readiness for Implementation | Learning Climate | Adoption (+)  Fidelity (+) | “I think between the two grade levels being able to work well together, it was easy to ask each other questions or even sometimes remind each other, ‘Hey, don't forget to log your kids' data for the week and things like that. Without hurting feelings, but just making sure that we're a team. So what we do, we do it together, and we do it well together.” |
|  | Leadership Engagement | Adoption (+)  Fidelity (+)  Penetration (+) | “I know our principal is really on board. She came to the conference at the beginning of the year. She really likes the program, as well as all of the ideas that it covers.”  “They were total, our principal was totally backing it. She was really embracing it. Before I had presented this to her, she was already working on a sensory pathway, getting that ordered and put together. So, she was already on top of wanting to increase movement and activity.” |
|  | Available Resources | Penetration (+)  Fidelity (+) | “I love that it's not a huge amount of time, but it does take time to get it implemented.”  “I know that resources are scarce but I have everything that I need to teach. I have the latest curriculum. I get a say in what we teach, which is nice… We have a big fundraiser and they were asking teachers to put in things for their wish list. What do you need? What do you want? And I'm hard-pressed to say I need anything more, at least right now.” |
|  | Self-Efficacy | Fidelity (+) | “Fairly confident. Worried about my first year and getting everything in, but, yeah, everyone was really helpful in sending out materials and asking if we had questions, and they were always there to answer them on time.” |
|  | Individual Stage of Change | Adoption (+) | “I would say it's very helpful, for me personally, to bring about diversity into your lesson. I have all kinds of plans of getting around and moving. One of my goals, and I don't meet it, and that makes me mad because it's a high goal that I have, is that I want to make learning more active.” |
| Individual Characteristics | Other Personal Attributes | Fidelity (+) | “But again, it comes down to the teacher who implemented it within her classroom. She was the one who ... And again, you have these people on all of your teams, you have the people who you can't put one more thing on their plate, and then you have others who are like, ‘Oh my gosh, I don't care what it takes. I'll be up to midnight. Let's do it.’” |
| Implementation Process | Planning | Fidelity (+) | “I was confident. I felt well going into it. The head of the dietary program and I were the ones that went to the training. I thought that went really well and felt comfortable leaving and ready to start it up again.”  “You guys had a document that was to use as a guide, and then we also used that note taking form, and we wrote our notes in there. I kept the notes, and then every team meeting I'd bring the notes with us.” |
|  | Engaging | Fidelity (+) | “Involving the students really helps a lot. Some of the students who were really excited about it and the teachers see that those students are excited about it and then also keeping it simple for some of those teachers because too much, they're just going to not want to do it.”  “Every time I would get something I would try to send that out. I did go every week and make sure we got the trinkets sent out. At the beginning, remember, I guess I went in and I gave a 20 minute presentation on how to do the website for the kids.” |
|  | Opinion Leader | Fidelity (+) | “The classroom teachers did a pretty nice job of trying to implement some things. They really took it on, but it really was kind of big.”  “We're both classroom teachers so our role was just implementing it within the classroom and supporting the other classroom teachers if they needed help. One of our other teachers had been through some of the training the year before, so she also was pretty helpful.” |
|  | Implementation Leaders | Adoption (+) | “When we look at the book, it's pretty straightforward on that. It's just finding the time to have, especially with the classroom teachers, to have them help implement and reinforce what we're teaching. We gave them posters and stuff.”  “I'm [leader], I'm the school nurse. I took coordination aspect of SWITCH and then recruited both [teachers] to help me with that. The three of us pretty much worked together. We did invite all the other teachers anytime we met, so that we could have everybody around the table for all the decision making. |
|  | Champions | Fidelity (+)  Adoption (+) | “So, our ‘at risk’ [counselor] and our guidance counselor were really curious to what the kids were logging in on because they were talking about it in middle school. And so, they were the ones, the first two that actually offered with family night that was scheduled to help out with anything with that too, and the SWITCH programs.” |
|  | Innovation Participants | Fidelity (+) | “We were starting to get results, better results with the students. Like they would report to me like, ‘Oh, I didn't use my phone at all.’ Or, ‘I'm only going to use it up until this certain hour.’ Like they had, they were able to vocalize their goals and report back on their goals a lot easier.”  “It's just I think, now, really, since we've done it three years, the kids just expect that we're doing it. Because that first year we bought pedometers, and they had a walking goal, and the last two years, I have parents saying, ‘Oh, the kids can't wait until they get to wear those pedometers.’ They think that's just part of what they're supposed to do.” |
|  | Executing | Fidelity (+)  Penetration (+) | “One of the things that we have to teach, like just switching what you ‘do’ part, I'm talking about sedentary lifestyle versus an active lifestyle and what that looks like in the short-term and long-term within their lifespan. It flows perfectly within health class.”  “And so, of course, implementation is pretty seamless because my staff know about the program, they know what to do now, they know what kind of data to take, those sorts of things. So, as far as implementation, from my standpoint, it was very good.” |
|  | Reflecting and Evaluating | Adoption (+)  Fidelity (+) | “We tried the folders this year with a specific lesson. She asked how that was going. I said, ‘Well, sometimes another teacher needs it at the same time.’ So then we used that feedback. She made two folders so two teachers could have the same lesson at the same time.”  “We keep making changes. We learn from our things and then we fix it and do it again, so I think we've done a good job. We found kind of what works in our school.” |

# Additional file 9: Overview of themes relating to sustainability

| **Sustainability Theme** | **Inexperienced Schools (n=17)** | **Experienced Schools (n=28)** |
| --- | --- | --- |
| **Importance of Student Awareness** | “Yeah. You know, maybe it's just more of an awareness, the kids have now become aware of they can change what they do, view, and chew kind of thing. Like carry with them. And that maybe next year when they see us in the hallway, it'll click and remember that kind of stuff.”  “I'd say they're pretty sustainable. I think that in particular the brain break side of things and the movement and the activity side of things is going to be the most applicable to everything. I'd say the one that was hardest for the kids was the view side of things. I don't know how sustainable that is. That one takes a lot more at home than what we see and can necessarily control. In the classroom, making sure that we're being more purposeful when we are using, like when they are on the computers. Is it something they could just be doing not on their computers? Is it an activity they have to be doing on their computers? Just being more purposeful in that sense. I think it's pretty sustainable though.”  “In PE, doing some of the new warmups and lesson plans, I think that's pretty sustainable. I can use those from year to year, look for new ideas each year, if they add new lesson plans next year. As far as that screen time content, keeping that going every year, I think that'll always be applicable, and we'll be able to use that, and going forward with lunchroom. I think it's all pretty sustainable.”  “Well I would say they're pretty highly sustainable. Making the changes in the lunchroom, getting the kids to buy in to trying the different fruits and vegetables, getting them moving more. Even just when we're the one on recess duty that we're... If we get this walking trail out there, the map out, it's just going to be that much easier to have that constant little reminder wherever they're at, that it's like, "Oh, make sure you're eating your fruits and vegetables." "Oh, make sure you're moving more." If we could get some pedometers and stuff for those kids, too, that are walking that, where they have that. Kids love gadgets.” | “After what we're all going through, gosh, I really hope so but I know for the fourth and fifth graders when I was that age if I had voluntary school, I could only imagine what sort of bad habits I might form. I don't know. And If mom and dad are working, it's really hard to keep those kids engaged and doing what they need to do I guess.”  “I think they'll be sustainable because if we continue to say, and I'm just going to focus in on the computer right now because this is the first year that 3rd, 4th, and 5th grade have their own Chromebooks, we've gone one-to-one, and to say, "No, these Chromebooks are for academics, not for gaming." And, again, I can only control what happens during the day. Now, those kids cannot take the computers home, they are assigned a certain computer and it's theirs through the plan, the plan is that they'll use them in middle school, they can't take them home.”  “We can continue to do the Do, View, Chew, continue to put that out. One thing I forgot to mention was we have like a TV screen that's like a sign board that goes and gives updates and things like that, and we would always put on there something about Do, View, and Chew.” |
| **Keeping it simple** | “Super easy, I mean, even the brain breaks because they talked about, Elani and Kendra, we can borrow that whenever, the kit that they gave us, so we can borrow that whenever. And then we've made copies of some of those things too, so that if we don't have access to it, we can still do some of those things. But I think it wasn't necessarily about all the stuff. It was more of the mindset and changing just what we're thinking. It doesn't take a lot of planning to add in a brain break, or it doesn't take a lot of planning to add in a lesson about eating healthy and those things because you can connect it to anything, any curriculum.”  “Of what we've done so far, I think very sustainable. I think truly as we think about SWITCH in the future and we also think about things that we're wanting to do to promote health and fitness, I think we're still at that infancy stage where it's only going to get bigger and better”  “I think building on it, is going to be part of it. Okay. And this is how I look at things because I have 30 years of being in business, and this is my frustration with being in education is that they try to change after one or two years, and I want to see if we can ... But at this point I have to say, we just have to continue going. You can ask me this question two years, three years, four years down the line.” | “I think the ones we made were pretty sustainable. We try to keep it simple. We didn't want to... It's hard. We've tried to do one thing at a time, to try and make sure, to see if it was going to work. So like, changing the milk, we can do that. We do that all the time, now. It's a lot easier. And the brain breaks in the classroom, that's sustainable. I think we've even had teachers at the kindergarten level start talking about it, because they're hearing about the benefits.”  “I will do that in the school setting in my classroom. I will continue to do those things I've done, like with the articles and the activity breaks, because you can tell when they get squirrel cigy you just like, okay, we have to do something now, get up, move to this side, if you think this, or to that side of the room, if you think... Not necessarily a complete exercise session, but just get up and move, yeah.” |
| **Integrating within school culture** | “I really see this as it's just part of our culture”  “I think they are very sustainable. I think since they just really focused it on the classroom piece, that piece will be more sustainable than anything, because they've done it once, so now they have a feel. Now that they know how the activities flow, and ... Oh, this really does only take 10 minutes of my day. Okay. Now, where can that 10 minutes fit in? And they can start to do it on a more consistent basis and figure out where it goes in their classroom routine.”  “Like I said about our kitchen, I know that that is sustainable. I mean, they are so onboard. It's so nice. And, that actually helps motivate a lot of things that we do because they are so onboard. It's like they made it part of their process and they made it part of their daily routine and they make it their vocabulary comes out, Switch-ish, and they're constantly talking with the kids about healthy choices. And so, I think it helps motivate everyone to go, "Oh, wait. They did this. Maybe if we made small changes, we could do this too."  “So, that's where we were going, and it seemed like everybody was on board. They wanted to continue and really embracing those things.” | “And some things might just become, day-to-day, what we do, and you don't even realize it, and, "Oh, that was because of SWITCH."  “I don't see anything changing. In fact, I don't remember if I said this before, but we're going to broaden our Switch committee to be a K-12 committee, and encourage it K-12. So, I feel like that will help us sustain it because it won't just be the PE teachers and myself and [team member] that are getting together and the nurse. We'll get some of those classroom teachers involved. And, I feel like the one thing that we could do better would be to take it outside the 12 weeks and not just, "Here's 12 weeks of SWITCH," but incorporate some activities throughout. We focus on movement a lot, but just making it more intentional, maybe.”  “I mean the brain breaks are definitely, since year one, I've kind of just done those now as part of what I've been doing in the classroom, as opposed to just waiting for Switch to start. And so that part's definitely sustainable.”  “I definitely think we'll continue. Even if we weren't doing SWITCH, we'll always have Wellness Wednesdays and Fitness Fridays. I'd like to see a little more follow through at lunchtime, but that's a work in progress and that has to do with a lot of different people.”  “Everything we did I think is sustainable. I think everything we did is only going to be able to be built on. We've documented everything we did so if anything happened to any of us it's ready to go for the next group of people. And our district has worked really hard over the last couple of years with trauma informed care and social/emotional learning, so SWITCH ties into that with activity breaks, things like that. I foresee that that's just become common practice for our teachers” |

# **
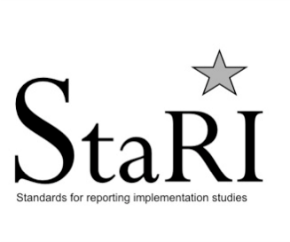
**Additional File 10: Standards for Reporting Implementation Studies: the StaRI checklist for completion

**Standards for Reporting Implementation Studies: the StaRI checklist for completion**

The StaRI standard should be referenced as: Pinnock H, Barwick M, Carpenter C, Eldridge S, Grandes G, Griffiths CJ, Rycroft-Malone J, Meissner P, Murray E, Patel A, Sheikh A, Taylor SJC for the StaRI Group. Standards for Reporting Implementation Studies [(StaRI) statement](http://www.bmj.com/content/356/bmj.i6795.full). *BMJ* 2017;356:i6795

The detailed Explanation and Elaboration document, which provides the rationale and exemplar text for all these items is: Pinnock H, Barwick M, Carpenter C, Eldridge S, Grandes G, Griffiths C, Rycroft-Malone J, Meissner P, Murray E, Patel A, Sheikh A, Taylor S, for the StaRI group. Standards for Reporting Implementation Studies [(StaRI). Explanation and Elaboration document](http://bmjopen.bmj.com/content/7/4/e013318.full?ijkey=vv4LKZxc25YcLJv&keytype=ref). *BMJ Open* 2017 2017;7:e013318

Notes: A key concept of the StaRI standards is the dual strands of describing, on the one hand, the implementation strategy and, on the other, the clinical, healthcare, or public health intervention that is being implemented. These strands are represented as two columns in the checklist.

| The primary focus of implementation science is the implementation strategy (column 1) and the expectation is that this will always be completed. | The evidence about the impact of the intervention on the targeted population should always be considered (column 2) and either health outcomes reported or robust evidence cited to support a known beneficial effect of the intervention on the health of individuals or populations. |
| --- | --- |

The StaRI standardsrefers to the broad range of study designs employed in implementation science. Authors should refer to other reporting standards for advice on reporting specific methodological features. Conversely, whilst all items are worthy of consideration, not all items will be applicable to, or feasible within every study.

| **Checklist item** | | **Reported on page #** | **Implementation Strategy** | **Reported on page #** | **Intervention** |
| --- | --- | --- | --- | --- | --- |
|  | |  | “Implementation strategy” refers to how the intervention was implemented |  | “Intervention” refers to the healthcare or public health intervention that is being implemented. |
| **Title and abstract** | | | | | |
| Title | **1** | 1 | Identification as an implementation study, and description of the methodology in the title and/or keywords | | |
| Abstract | **2** | 2 | Identification as an implementation study, including a description of the implementation strategy to be tested, the evidence-based intervention being implemented, and defining the key implementation and health outcomes. | | |
| **Introduction** | | | | | |
| Introduction | **3** | 4 | Description of the problem, challenge or deficiency in healthcare or public health that the intervention being implemented aims to address. | | |
| Rationale | **4** | 4-6 | The scientific background and rationale for the implementation strategy (including any underpinning theory/framework/model, how it is expected to achieve its effects and any pilot work). | 5 | The scientific background and rationale for the intervention being implemented (including evidence about its effectiveness and how it is expected to achieve its effects). |
| Aims and objectives | **5** | 6-7 | The aims of the study, differentiating between implementation objectives and any intervention objectives. | | |
| **Methods: description** | | | | | |
| Design | **6** | 7 | The design and key features of the evaluation, (cross referencing to any appropriate methodology reporting standards) and any changes to study protocol, with reasons | | |
| Context | **7** | 7-8 | The context in which the intervention was implemented. (Consider social, economic, policy, healthcare, organisational barriers and facilitators that might influence implementation elsewhere). | | |
| Targeted ‘sites’ | **8** |  | The characteristics of the targeted ‘site(s)’ (e.g locations/personnel/resources etc.) for implementation and any eligibility criteria. | 7-8 | The population targeted by the intervention and any eligibility criteria. |
| Description | **9** |  | A description of the implementation strategy | 7-8 | A description of the intervention |
| Sub-groups | **10** | N/A | Any sub-groups recruited for additional research tasks, and/or nested studies are described | | |
| **Methods: evaluation** | | | | | |
| Outcomes | **11** |  | Defined pre-specified primary and other outcome(s) of the implementation strategy, and how they were assessed. Document any pre-determined targets | 8-9 | Defined pre-specified primary and other outcome(s) of the intervention (if assessed), and how they were assessed. Document any pre-determined targets |
| Process evaluation | **12** | 9-11 | Process evaluation objectives and outcomes related to the mechanism by which the strategy is expected to work | | |
| Economic evaluation | **13** | N/A | Methods for resource use, costs, economic outcomes and analysis for the implementation strategy |  | Methods for resource use, costs, economic outcomes and analysis for the intervention |
| Sample size | **14** | 8 | Rationale for sample sizes (including sample size calculations, budgetary constraints, practical considerations, data saturation, as appropriate) | | |
| Analysis | **15** | 11-14 | Methods of analysis (with reasons for that choice) | | |
| Sub-group analyses | **16** | 13-14 | Any a priori sub-group analyses (e.g. between different sites in a multicentre study, different clinical or demographic populations), and sub-groups recruited to specific nested research tasks | | |

| **Results** | | | | | |
| --- | --- | --- | --- | --- | --- |
| Characteristics | **17** | N/A | Proportion recruited and characteristics of the recipient population for the implementation strategy |  | Proportion recruited and characteristics (if appropriate) of the recipient population for the intervention |
| Outcomes | **18** |  | Primary and other outcome(s) of the implementation strategy | 14-16 | Primary and other outcome(s) of the Intervention (if assessed) |
| Process outcomes | **19** | N/A | Process data related to the implementation strategy mapped to the mechanism by which the strategy is expected to work | | |
| Economic evaluation | **20** | N/A | Resource use, costs, economic outcomes and analysis for the implementation strategy |  | Resource use, costs, economic outcomes and analysis for the intervention |
| Sub-group analyses | **21** | N/A | Representativeness and outcomes of subgroups including those recruited to specific research tasks | | |
| Fidelity/ adaptation | **22** |  | Fidelity to implementation strategy as planned and adaptation to suit context and preferences | 13-14 | Fidelity to delivering the core components of intervention (where measured) |
| Contextual changes | **23** | 14-17 | Contextual changes (if any) which may have affected outcomes | | |
| Harms | **24** | N/A | All important harms or unintended effects in each group | | |
| **Discussion** | | | | | |
| Structured discussion | **25** | 19 | Summary of findings, strengths and limitations, comparisons with other studies, conclusions and implications | | |
| Implications | **26** |  | Discussion of policy, practice and/or research implications of the implementation strategy (specifically including scalability) | 24 | Discussion of policy, practice and/or research implications of the intervention (specifically including sustainability) |
| **General** | | | | | |
| Statements | **27** | 26 | Include statement(s) on regulatory approvals (including, as appropriate, ethical approval, confidential use of routine data, governance approval), trial/study registration (availability of protocol), funding and conflicts of interest | | |
